# Development of a mitochondrial mini-barcode and its application in metabarcoding for identification of leech in traditional Chinese medicine

**DOI:** 10.1038/s41598-024-77913-6

**Published:** 2025-01-11

**Authors:** Chenfeng Shi, Yuhan Guo, Lijuan Yao, Yunhui Xu, Jing Zhou, Moli Hua

**Affiliations:** National Key Laboratory of Lead Druggability Research, Shanghai Institute of Pharmaceutical Industry, State Institute of Pharmaceutical Industry, 201203 Shanghai, People’s Republic of China

**Keywords:** Leech, Metabarcoding, Identification, Mini-barcode, Genetic markers, Phylogenetics, Medical research

## Abstract

In Traditional Chinese Medicine (TCM), the medicinal leech is vital for treatments to promote blood circulation and eliminate blood stasis. However, the prevalence of counterfeit leech products in the market undermines the quality and efficacy of these remedies. Traditional DNA barcoding techniques, such as the *COI* barcode, have been limited in their application due to amplification challenges. This study identified high variability in the *16 S rRNA* gene within the mitochondrial genome across five leech species, leading to the development of a novel 219 bp mini-barcode. Compared with the traditional *COI* barcode, our mini-barcode showed remarkable identification efficiency, classifying 142 out of 147 leech samples from fresh and processed materials. In contrast, the *COI* barcode could only successfully identify 79 out of the 147 samples. In the case of seven batches of leech decoction pieces, the mini-barcode identified six, whereas the *COI* barcode only recognized one. Additionally, the mini-barcode effectively discerned five leech species within Chinese patent medicines when combined with metabarcoding technology. These results confirm the mini-barcode’s potential as a reliable tool for rapidly and precisely identifying leech species in TCM products.

**Supplementary Information:**

The online version contains supplementary material available at 10.1038/s41598-024-77913-6.

## Introduction

The medicinal leech is a crucial remedy for promoting blood circulation and dispelling blood stasis, was first recorded in *Shen Nong Ben Cao Jing*, known as “shuizhi” or “mahuang”^1^. Modern pharmacological studies have demonstrated its multifaceted effects, including anticoagulant^2^, anti-platelet aggregation^3^, anti-inflammatory^4^, and regulation of lipid metabolism^5^. The Pharmacopoeia of the People’s Republic of China includes three leech species, namely *Whitmania pigra* (WP), *Hirudo nipponia* (HN), and *Whitmania acranulata* (WA), for medicinal purposes^6^. Because of the colloquial terms “shuizhi” or “mahuang” for the annelids of the Hirudinea class in China, coupled with their morphological similarities, numerous counterfeits have emerged, such as *Poecilobdella manillensis* (PM), *Whitmania laevis* (WL), and *Erpobdella* sp. (Er). The source of the species plays a decisive role in the chemical composition of leechs^7^. Therefore, the accurate identification of leech origins is particularly important.

The traditional methods for the identification of medicinal leech materials include morphological analysis^8^ and chemical identification^9^, but these methods depend on experience, have strong subjectivity, and are hard to distinguish between processed products^10^. DNA barcoding is a technique for authenticating species using a standard DNA region and has made remarkable progress in species identification^11^. The *COI* gene is widely used in the identification of animals as a universal DNA barcode^12,13^. However, universal DNA barcoding has certain limitations: it is incapable of identifying the species of herbal materials in Chinese patent medicine (CPM) and proprietary Chinese medicinal materials that have undergone severe DNA degradation^14^. Unfortunately, the medicinal material of leeches typically undergoes drying or high-temperature processing during preparation, which leads to DNA degradation. Furthermore, it is often used in conjunction with other medicinal materials in the formulation of CPM. The emergence of DNA metabarcoding technology may be able to solve this problem.

DNA metabarcoding is a novel approach that identifies multiple species from a mixed sample based on high-throughput sequencing of a specific DNA marker^15^. High-throughput sequencing generates DNA sequence data, which can be used to identify many species present in a sample through bioinformatics analysis^16^. In theory, the proportion of species reads obtained from a mixed sample should be directly proportional to the abundance of the species within the sample^17^. Therefore, species reads obtained through DNA metabarcoding can reflect the richness of species in the community to a certain extent. Nowadays, DNA metabarcoding is also used for the identification of herbal species in CPM^18–20^. However, the selection of barcodes for DNA metabarcoding remains a challenge. Conventional barcodes, such as *COI*, *ITS2*, *matK*, *rbcL*, and *trnH-psbA*, generally longer than 500 bp, which poses considerable difficulties for PCR amplification in the case of degraded DNA from herbal materials and CPM^21^. Barcodes that are too short may lack sufficient resolution to differentiate closely related species due to their abbreviated length, e.g., the P6 loop of *trnL* intron^22^. Consequently, it is crucial to identify a concise yet information-rich DNA barcode, which is referred to as a mini-barcode.

The DNA mini-barcode is a fragment of DNA ranging from 100 to 250 bp in length that possesses sufficient variable sites for species identification^23^. Due to the significant reduction in the length of the barcode, the success rate of PCR amplification is greatly increased. However, the resolution of the barcode is consequently diminished. Therefore, it is necessary to design specific mini-barcodes for closely related species. For animals, developing specific DNA mini-barcodes from the mitochondrial genome is a straightforward and feasible approach^24^. The majority of animal mitochondrial genomes are compact and circular in structure, ranging between 16,000 and 20,000 base pairs in length, devoid of introns, and their fundamental structural and functional regions are usually highly conserved^25^. Due to the mitochondrial genome’s characteristics of maternal inheritance, multiple copies, and rapid evolution, it is widely used in the development of molecular markers^26,27^. Additionally, the rich interspecific variability of the mitochondrial genome makes it a useful tool for providing high-resolution barcodes for closely related species^28^. The practicality of mini-barcodes has been validated in many specific groups, such as marine animals^29^, plankton^30^, wildlife^31^, and processed Chinese medicine products^32^.

In the present study, we developed a specific mini-barcode derived from mitochondrial genomes and combined it with metabarcoding technology for the identification of medicinal leech species in CPM. The aims of our study included: (1) to develop a mini-barcode for species identification of five leech species; (2) to compare the applicability and accuracy of the mini-barcode and *COI* gene in identifying leeches; (3) to test the feasibility of combining mini-barcode and DNA metabarcoding techniques for identification of CPM. Our results will provide a basis for accurately distinguishing medicinal leeches, ensuring the quality of medicinal materials, and offering guidance for the quality control of other animal-based medicines.

## Results

### Complete mitochondrial genome features of five leech species

**Fig. 1:**
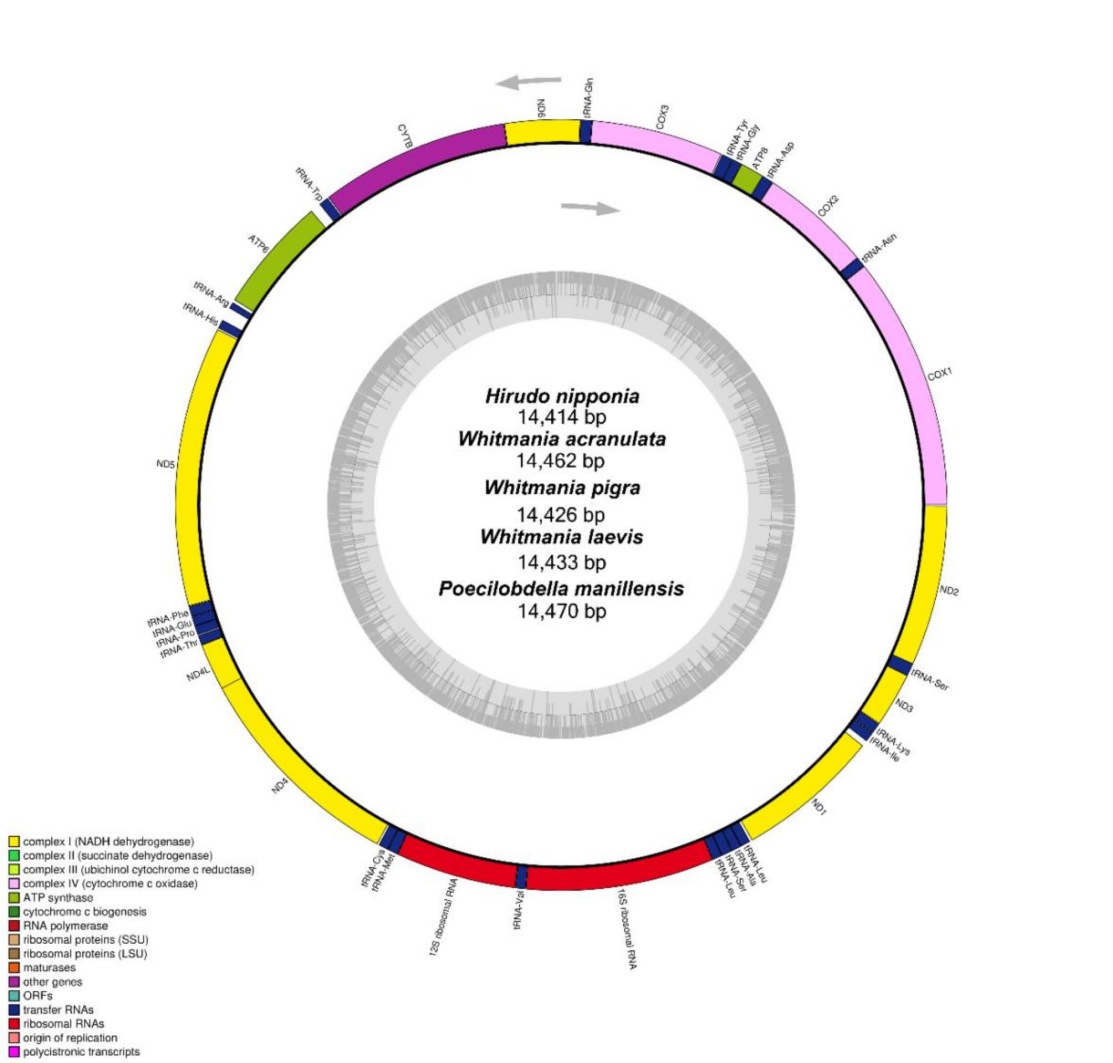
Gene map of the complete mitochondrial genomes of the five leech species. Genes annotated on the outer circle are encoded on the positive strand (J-strand), while those annotated on the inner circle are encoded on the negative strand (N-strand). The dark gray and light gray shading within the inner circle correspond to the percentages of G + C and A + T contents, respectively.

As shown in Fig. 1, the size of the mitochondrial genomes in five leech species ranges from 14,414 bp to 14,470 bp, and their structures are similar (Table 1), featuring circular molecules with 13 protein-coding genes, including those encoding the cytochrome c oxidase subunits (*cox*1-3), ATP synthase subunits (*ATP6* and *ATP8*), NADH dehydrogenase subunits (*ND*1-6 and *ND4L*), and the cytochrome b deoxyribonucleotide gene (*Cytb*). Additionally, there are 22 tRNA genes (*tRNA-Gln*, *tRNA-Tyr*, *tRNA-Gly*, *tRNA-Asp*, *tRNA-Asn*, *tRNA-Ser*, *tRNA-Lys*, *tRNA-Ile*, *tRNA-Lue*, *tRNA-Ala*, *tRNA-Ser*, *tRNA-Leu*, *tRNA-Val*, *tRNA-Met*, *tRNA-Cys*, *tRNA-Thr*, *tRNA-Pro*, *tRNA-Glu*, *tRNA-Phe*, *tRNA-His*, *tRNA-Arg*, *tRNA-Trp*) and 2 rRNA genes (*16 S rRNA* and *12 S rRNA*). The GC content of their mitochondrial genomes ranges from 27.4 to 28.4%. All genes are encoded on the positive strand (J-strand).

**Table 1:** Comparison of the mitochondrial genome organization of the five leech species.

Genome features	Hirudo nipponia	Whitmania acranulata	Whitmania laevis	Whitmania pigra	Poecilobdella manillensis
Total reads (bp)	14,414	14,462	14,433	14,426	14,470
Number of genes	37	37	37	37	37
Protein-coding genes	13	13	13	13	13
tRNA genes	22	22	22	22	22
rRNA genes	2	2	2	2	2
Total G + C content (%)	27.4	28.4	28.1	27.8	28.0

### Comparison of the mitochondrial genome structures of the five leech species

**Fig. 2:**
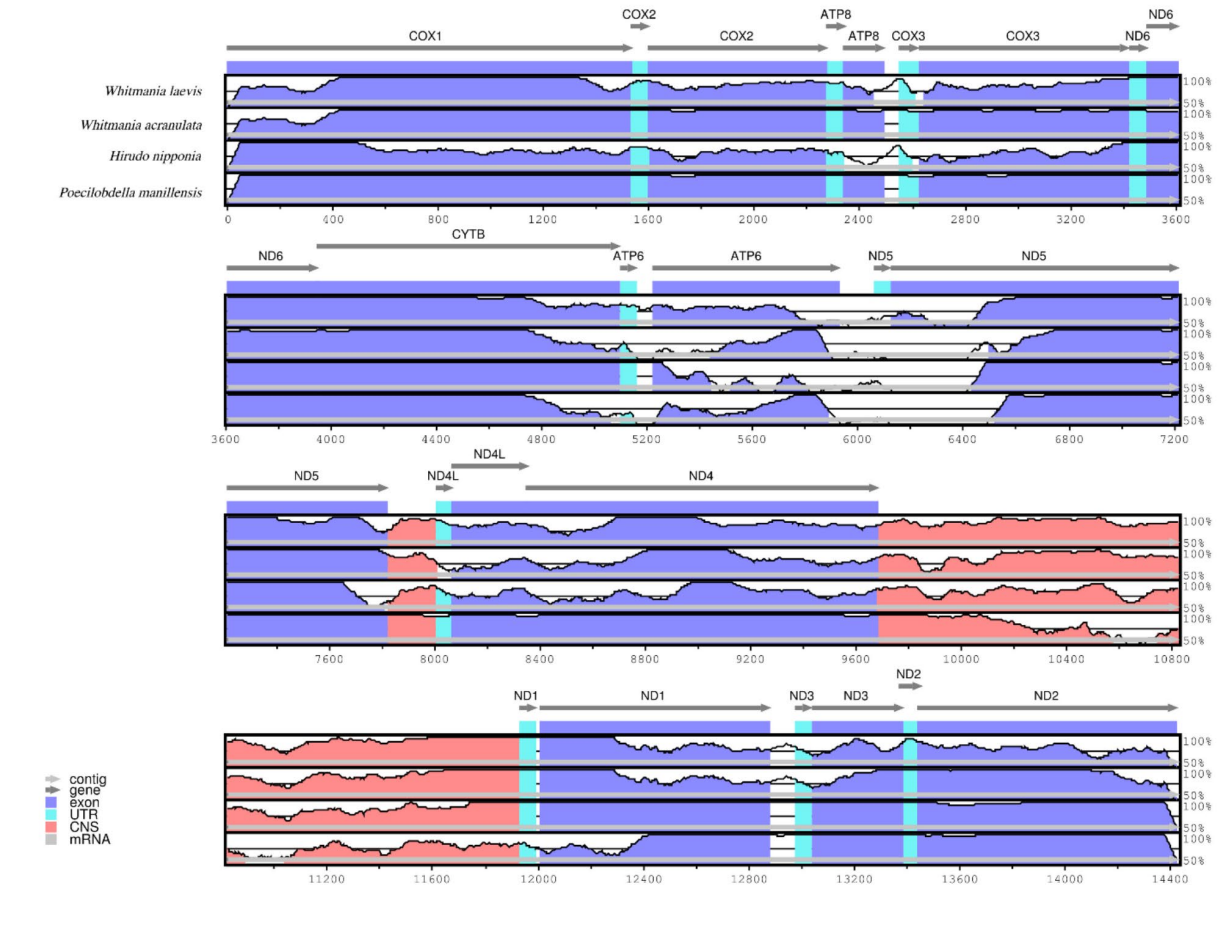
Visual alignment of the mitochondrial genomes of the five leech species. VISTA-based identity plot shows the sequence identity of the five species, using *W. pigra* as a reference.

Multiple sequence alignment of the mitochondrial genomes of the five leech species was performed by mVISTA, using the annotated mitochondrial genome sequence of *W. pigra* as reference. The result (Fig. 2) showed that the genomes of the five species are highly conserved, with some degree of divergence, and most of these highly variable regions were observed in conserved noncoding sequences (CNS). Comparative analysis by MAUVE showed that the mitochondrial genome structures of the five leech species were identical (Supplementary Figure S1).

### Selection of mini-barcode and design of primers

**Fig. 3:**
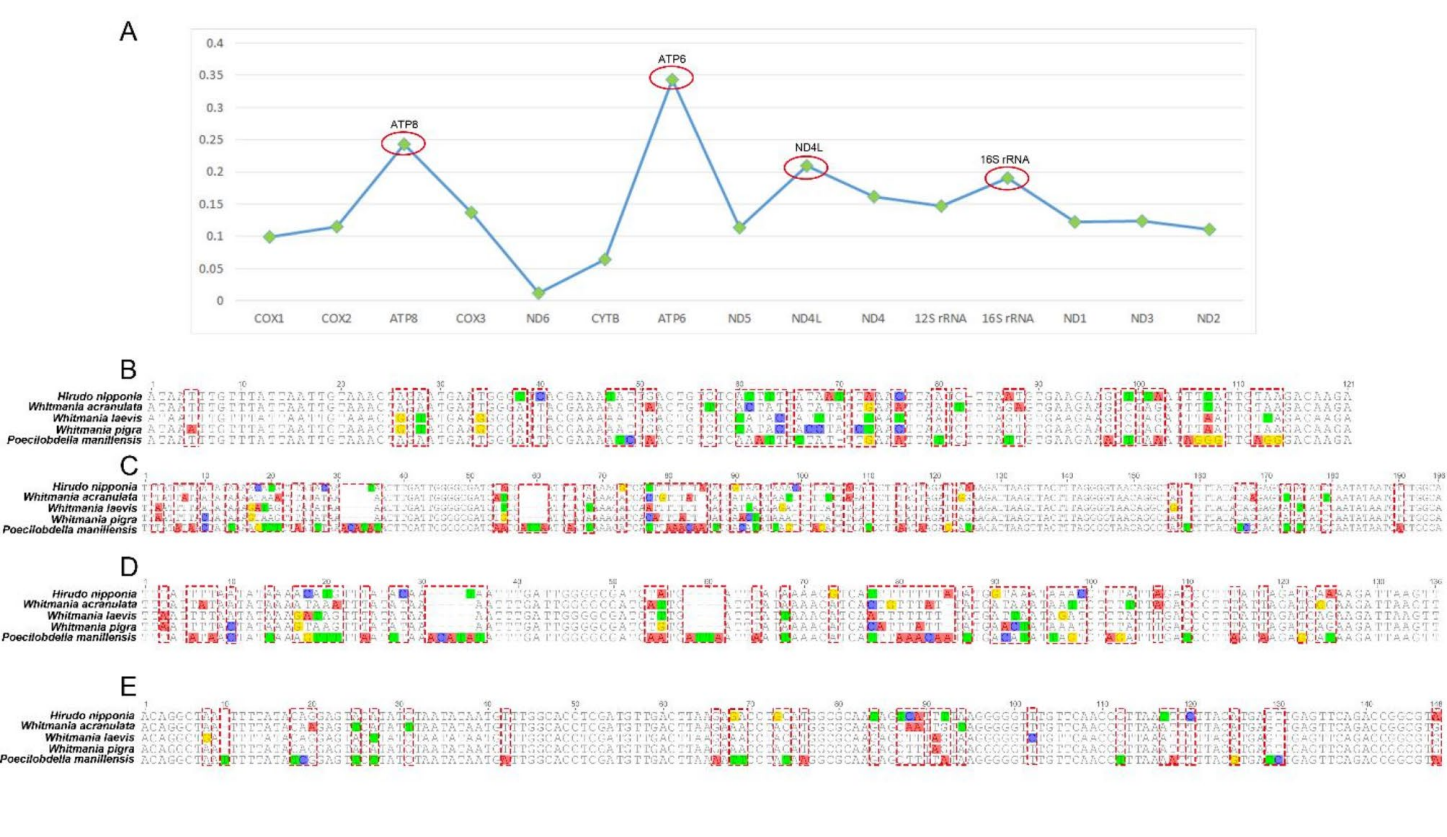
The selection of hypervariable coding regions and examination of variable sites in four mini-barcodes, red dotted frames present variable sites, conserved bases among the five leech species are shown in black, while divergent bases are indicated in different colors. (A) The Pi values of the shared 15 genes of five mitochondrial genomes (B) The mini-barcode of primer 606 F/744R (C) The mini-barcode of primer 741 F/943R (D) The mini-barcode of primer 742 F/884R (E) The mini-barcode of primer 876 F/1043R.

**Fig. 4:**
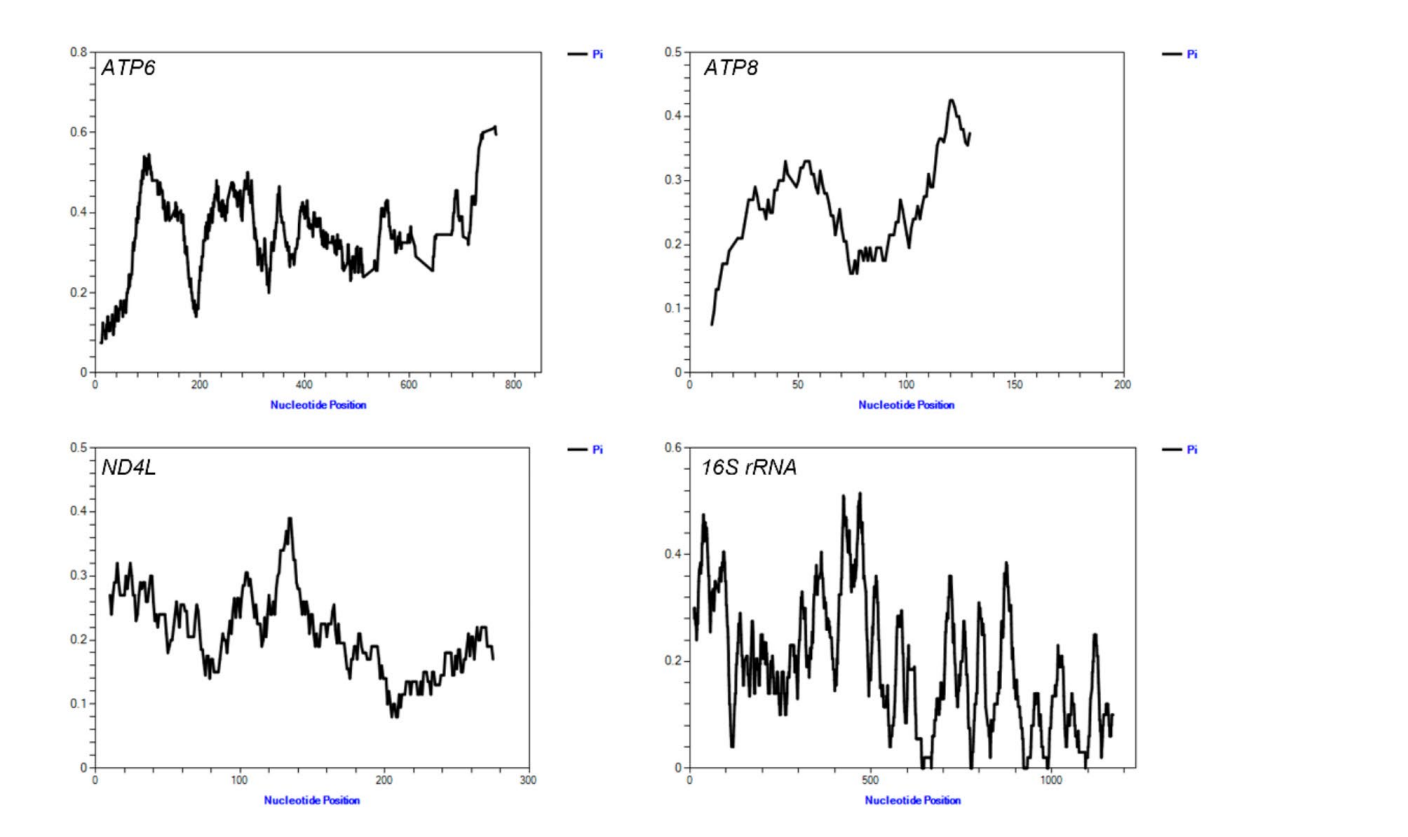
Sliding window analysis of nucleotide variability in the *ATP6*, *ATP8*, *ND4L*, and *16 S rRNA* genes.

Highly variable DNA regions of mitochondrial genomes could be used to distinguish between closely related species. In this study, a total of 15 genes shared among the five leech species were used to estimate nucleotide diversity. The results showed that the nucleotide variability (Pi) of a shared gene among the five species ranged from 0.0115 to 0.3433 (Fig. 3A). The *ATP6* region showed the highest levels of divergence (0.3433), followed by *ATP8* (0.2424), *ND4L* (0.2091), and *16 S rRNA* (0.1901). To ensure that the developed primers could amplify across multiple leech species, a sliding window analysis (with a window size of 20 bp and a step size of 1 bp) was conducted to calculate the Pi values for the *ATP6*, *ATP8*, *ND4L*, and *16 S rRNA* genes. The results revealed that only the *16 S rRNA* gene contained regions with a Pi value of 0, which are suitable for primer design (Fig. 4). Consequently, the 16 S rRNA gene was selected for the development of the mini-barcode.

Four primer pairs were designed within the conservative region in *16 S rRNA* using Primer3^33^ (Table 2). After removing the primer regions, the amplicon sizes for primers 606 F/744R, 741 F/943R, 742 F/884R, and 876 F/1043R are 121, 196, 136, and 151 bp, respectively (Fig. 3B, C, D, E). The mini-barcode of primer 741 F/943R had 55 variable sites, which is higher than others. Considering the length of the barcode region and the physicochemical properties of the primers, 741 F/943R was selected as the primer of the mini-barcode.

**Table 2:** Primers developed from *16 S rRNA*.

Primer name	primer sequence 5’ to 3’	Sequence excluding primers (bp)	Variable sites of mini-barcode	GC%	Tm
606 F	TGTGCAAAGGTAGCATA	121	38	41.2	49.0
744R	AAGCTTTATAGGGTCT			37.5	46.9
741 F	AGAAGACCCTATAAAGC	196	55	41.2	48.6
943R	TTAAGTCAACATCGAGG			41.2	47.9
742 F	GAAGACCCTATAAAGC	136	47	43.8	45.3
884R	CTGTTACCCCTAAAGT			43.8	46.4
876 F	ATTAAGTTACTTTAGGGGTA	148	30	30.0	48.6
1043R	TAGAATCTAACCTGGCT			41.2	47.9

### Morphological identification and typical characteristics of leeches

**Fig. 5:**
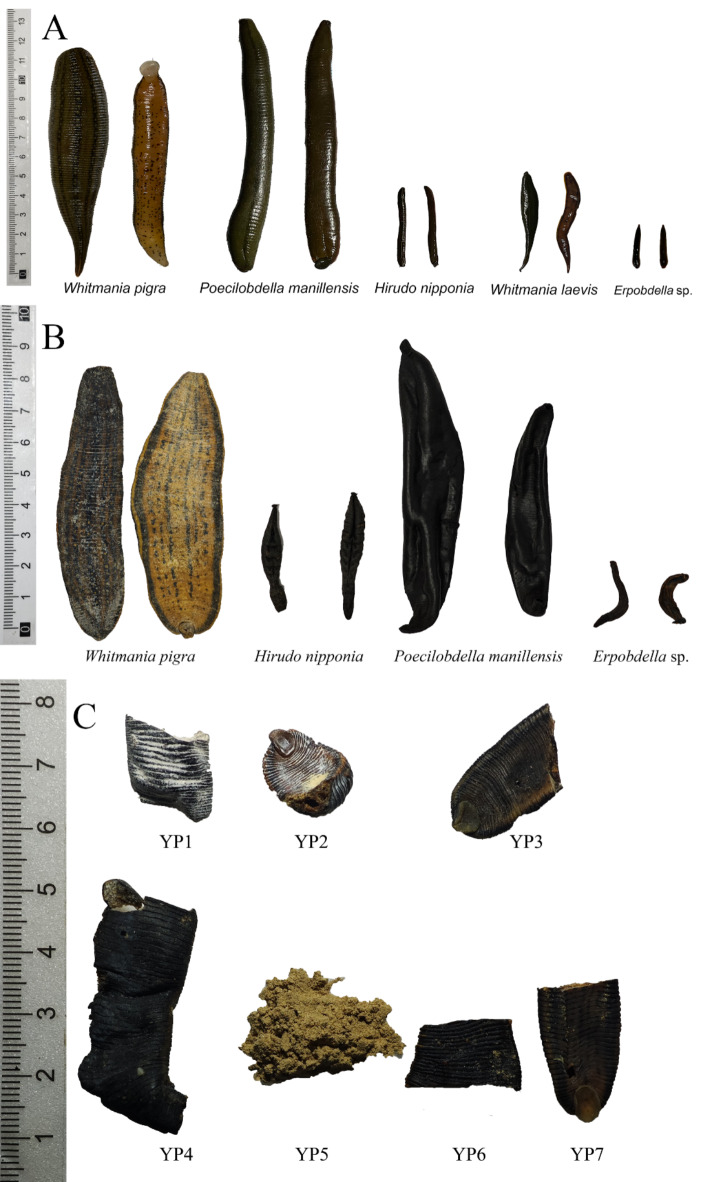
Typical characteristics of leeches: (A) Fresh samples; (B) Medicinal material samples; (C) Herbal decoction pieces samples.

All samples were subjected to morphological identification to determine their species, with the results presented in Table 3. The typical morphological characteristics of the samples are shown in Fig. 5.

*Whitmania pigra* is relatively large, with a strong ability to contract and extend. When fully extended, it is almost linear, while in a fully contracted state, it becomes nearly spherical. Its length can reach 10–15 cm when extended and 4–5 cm when contracted. The dorsal side is dark brown with five distinct black stripes running from head to tail, featuring spaced white spots along the stripes. The caudal sucker is relatively large. The ventral side is pale yellow with numerous black spots, some of which may align into linear patterns along the midline. Additionally, a yellow line runs from head to tail on each side. The dried medicinal material is hard, flexible, and not easily broken, with a broad body and more pronounced abdominal characteristics. Photos of samples can be found in Supplementary Figure S2 and Figure S6.

*Hirudo nipponia* is relatively small, with a slender, somewhat cylindrical shape and moderate extensibility. The head is narrower, while the mid to posterior segments are thicker. When extended, its length is approximately 3–10 cm, with a width of less than 1 cm. Both the dorsal and ventral sides are black, with five white stripes on the dorsal side, the central stripe being the most distinct. In the dried medicinal material, the head remains narrow, while the mid to posterior segments are thicker. The stripes are nearly invisible, and the texture is hard and brittle, making it prone to breaking. The entire specimen is black, and some samples may reveal dry blood clots from the host when broken. Photos of samples can be found in Supplementary Figure S3 and Figure S7.

*Poecilobdella manillensis* is relatively large, with a length exceeding 10 cm. Its extensibility is less than that of *Whitmania pigra*. The dorsal side is dark green with a black stripe running from head to tail, which is free of spots. The sides have yellow lines extending from head to tail, and the dorsal side adjacent to these lines features black spots. The ventral side is dark brown and lacks spots. The dried medicinal material is hard and brittle, making it prone to breaking. Both the dorsal and ventral sides are black, and some samples may reveal dry blood clots from the host when broken. Photos of samples can be found in Supplementary Figure S4 and Figure S8.

*Whitmania laevis* resembles *Whitmania pigra* in shape but is smaller in size. The body is slightly spindle-shaped, tapering gradually towards the front and widening and rounding at the rear, with minimal change in width towards the posterior half. The caudal sucker is relatively small. The body length typically ranges from 3 to 8 cm, and the width from 0.5 to 1.5 cm. The dorsal side is usually olive green to brown with five longitudinal yellow stripes. Dried specimens of this species were not collected. Photos of samples can be found in Supplementary Figure S5.

*Erpobdella* sp. is relatively small, with a length of approximately 2 to 4 cm and a width of less than 1 cm. Its morphology is similar to that of *Hirudo nipponia*, but with less extensibility. The dorsal side lacks white stripes, and the body is slender and elongated, tapering gradually towards the front while maintaining a relatively constant width towards the rear. Both the dorsal and ventral sides are black. The dried specimens are small and curved, and are prone to breaking. Since this species does not feed on blood, there are no dry blood clots from the host observed in the broken specimens. Photos of samples can be found in Supplementary Figure S5 and Figure S9.

### The efficiency evaluation of using the 741 F/943R primer pair for mini-barcode identification in fresh leeches, medicinal materials, and decoction pieces

To assess the identification efficiency of the mini-barcode primer 741 F/943R, we concurrently amplified the universal *COI* barcode using primers LCO1490/HCO2198 for comparison. PCR amplification was performed on 147 leech samples using both primer pairs, comprising 77 fresh leeches and 70 medicinal material leech samples (Table 3). All samples were identified to the species or genus level based on morphological characteristics. Sequences were obtained after Sanger sequencing and assembly.

**Fig. 6:**
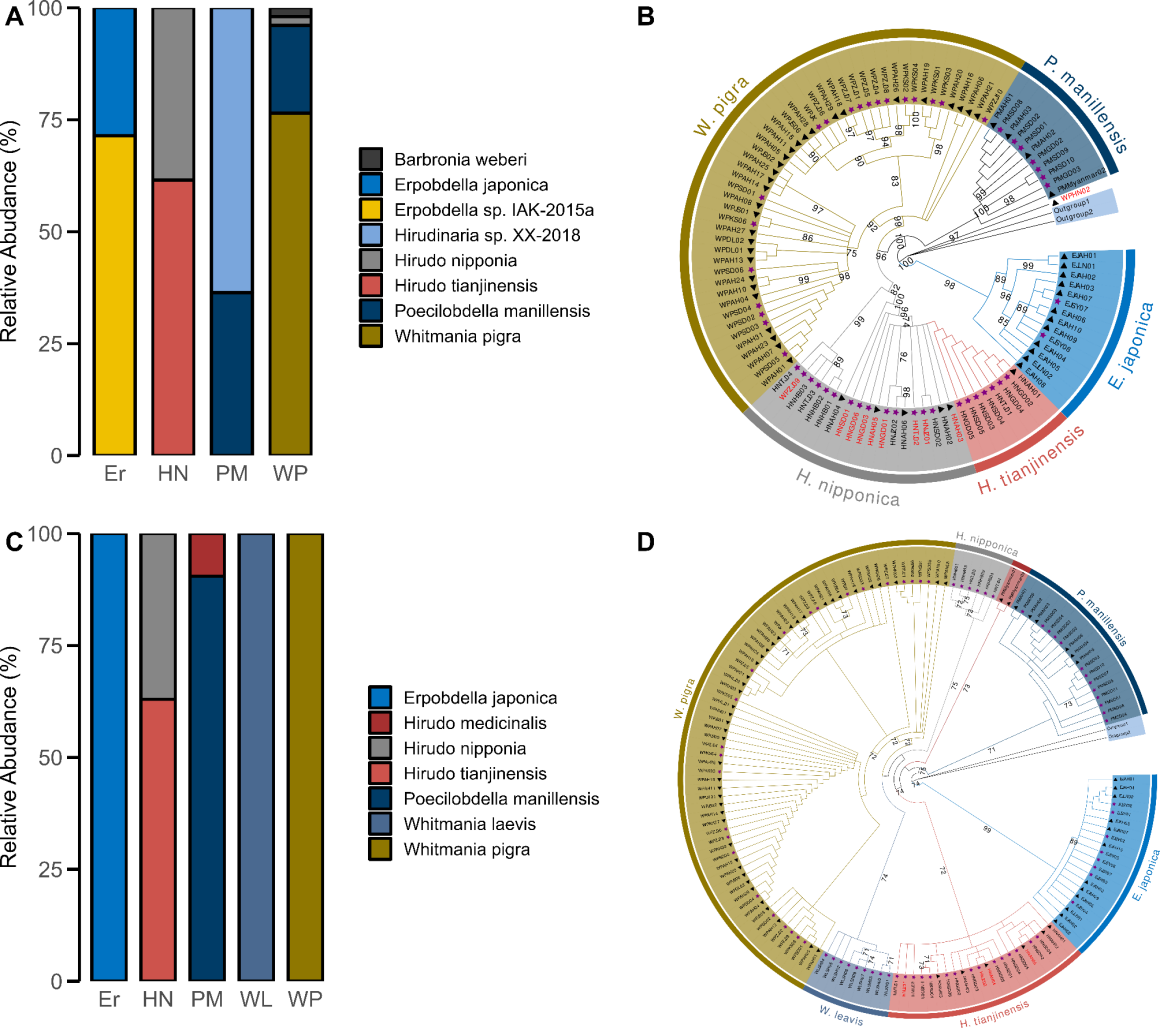
Identification results and Phylogenetic tree of fresh leeches and medicinal leech specimens, the pentagon represents fresh samples, while the triangle represents medicinal material samples, samples marked in red indicate discrepancies between the BLAST results and the phylogenetic tree results. (A) BLAST result of COI . (B) Phylogenetic tree of COI. (C) BLAST result of mini-barcode. (D) Phylogenetic tree of mini-barcode. Er: *Erpobdella sp.*; HN: *Hirudo nipponia*; PM: *Poecilobdella manillensis*; WL: *Whitmania laevis*; WP: *Whitmania pigra*.

As a result, 102 sequences were obtained using LCO1490/HCO2198, with a success rate of 70%. As shown in Fig. 6A, the samples in the Er group were identified as *Erpobdella japonica* and *Erpobdella* sp. IAK-2015a (unclassified *Erpobdella*). Due to insufficient morphological characteristics, we consider these identification results to be valid. The HN group included samples identified as *Hirudo tianjinensis*, a species first described by Wang et al. in Tianjin, which closely resembles HN morphologically^34^. Therefore, it is possible that some of the collected samples were *Hirudo tianjinensis* but were misidentified as *Hirudo nipponia*. Seven samples in the PM group were identified as *Hirudinaria* sp. XX-2018 (unclassified *Hirudinaria*). However, we confirm that the collected samples are *Poecilobdella manillensis* due to their distinct longitudinal stripes and orange-yellow stripes on the sides. In the WP group, ten samples were identified as *Poecilobdella manillensis* and one as *Hirudo nipponia*, both of which are blood-feeding leeches and can be easily distinguished from *Whitmania pigra*. One sample was identified as *Barbronia weberi*, but *Whitmania pigra* has notable differences in size and stripes. Thus, the twelve samples in the WP group were misidentified. The BLAST results of *COI* are presented in Supplementary Table S1. A phylogenetic tree was constructed using 102 and 2 outgroup sequences (Fig. 6B). The results showed that, for most species, each sample was clustered into its clade, except for WPZJ09 and WPHN02. Eight samples identified as *Hirudo tianjinensis* clustered together, while eight samples within the *H. nipponica* cluster were still identified as *Hirudo tianjinensis*.

After amplifying using 741 F/943R, the sequences of WLSH05, HNSD06, and WPAH02 were not successfully sequenced, and the success rate was 98%. As shown in Fig. 6C, all samples in the Er group were identified as *Erpobdella japonica*. Although identification was achieved at the species level, we cannot yet determine its accuracy. In the HN group, 17 samples were identified as *Hirudo tianjinensis*, further indicating the possibility of errors in morphological identification. PMMyanmar01-02 was identified as *Hirudo medicinalis*, but due to drying, the patterns and other morphological characteristics have become less distinct, complicating the accuracy of this identification. The BLAST results for WL and WP were consistent with the morphological identification. The BLAST results of 741 F/943R are provided in Supplementary Table S2. A phylogenetic tree was constructed using 144 and 2 outgroup sequences (Fig. 6D). The samples in the HN group were divided into two clusters: *H. tianjinensis* and *H. nipponica*. Except for four samples identified as *Hirudo nipponica* that were placed in the *H. tianjinensis* cluster, the remaining samples showed consistency between their BLAST results and the phylogenetic tree results.

In summary, *COI* can successfully identify some samples, but 30% of the samples, such as WL, failed to yield *COI* sequences. Additionally, contamination from host DNA complicates obtaining high-quality sequencing results for samples in the HN and PM groups. The misidentification of samples in the WP group may be due to DNA degradation in dried samples, leading to low-quality sequencing results. The primers 741 F/943R provide sufficient variability to distinguish between different leech species. More importantly, they are easier to amplify compared to *COI* and are not affected by host DNA contamination.

Seven batches of leech herbal decoction pieces were collected to evaluate the identification capability of 741 F/943R for processed medicinal materials. All samples were identified as WP based on morphological characteristics, except for YP5, which had already been ground into powder. Using LCO1490/HCO2198, the *COI* segments of four decoction piece samples were successfully amplified, sequenced, and assembled. The resulting sequences were subjected to a BLAST analysis, revealing that YP3 was identified as PM, which are blood-feeding leeches and can be easily distinguished from *Whitmania pigra*.YP6 was identified as *Erpobdella octoculata*. Leeches of the genus *Erpobdella* typically measure below 70 mm in length, making them easily distinguishable from *Whitmania pigra*. Therefore, we consider this to be an incorrect identification result. YP4 and YP7 were identified as WP. 741 F/943R successfully amplified mini-barcode segments from seven samples. Following sequencing and assembly, sequences for all samples except YP1 were obtained. BLAST results indicated that these six samples were identified as WP. The sample information and identification results are shown in Table 4. The results suggest that the *COI* barcode could only successfully identify two herbal decoction piece sample, while the mini-barcode successfully identified six samples. This demonstrates that the mini-barcode we developed has a more significant advantage in severely degraded DNA samples.

**Table 3:** Samples of fresh leeches and medicinal material are used to assess the efficiency of the mini-barcode.

Sample type	Morphological identification	Location	Number of samples	Voucher No.	Group
Fresh	*W. pigra*	Jiangsu	6	WPKS01-06	WP
	*W. pigra*	Jiangxi	1	WPJX	
	*W. pigra*	Zhejiang	10	WPZJ01-10	
	*W. pigra*	Shanghai	1	WPSH	
	*W. pigra*	Shandong	6	WPSD01-06	
	*H. nipponica*	Shandong	6	HNSD01-06	HN
	*H. nipponica*	Hebei	4	HNHB01-04	
	*H. nipponica*	Tianjin	4	HNTJ01-04	
	*H. nipponica*	Guangdong	6	HNGD01-06	
	*H. nipponica*	Hubei	2	HNJZ01-02	
	*P. manillensis*	Guangdong	3	PMGD01-03	PM
	*P. manillensis*	Yunnan	10	PMYN01-10	
	*W. leavis*	Shanghai	10	WLSH01-10	WL
	*Erpobdella* sp.	Liaoning	8	ErSY01-08	Er
Medicinal material	*W. pigra*	Anhui	32	WPAH01-32	WP
	*W. pigra*	Hunan	2	WPHN01-02	
	*W. pigra*	Jiangsu	6	WPJS01-06	
	*W. pigra*	Heilongjiang	2	WPHLJ01-02	
	*W. pigra*	Liaoning	2	WPDL01-02	
	*H. nipponica*	Anhui	6	HNAH01-06	HN
	*P. manillensis*	Anhui	6	PMAH01-06	PM
	*P. manillensis*	Myanmar	2	PMMyanmar01-02	
	*Erpobdella* sp.	Anhui	10	ErAH01-10	Er
	*Erpobdella* sp.	Liaoning	2	EJLN01-02	

**Table 4:** The sample information and identification results of leech herbal decoction pieces.

	Processing methods	Location	Morphological identification	Identification results	
				LCO1490/HCO2198	741 F/943R
YP1	Scalding with Boiling Water	Shandong	*W. pigra*	/	/
YP2		Shandong	*W. pigra*	/	WP
YP6		Shandong	*W. pigra*	EO	WP
YP3	Hang-drying	Shandong	*W. pigra*	PM	WP
YP4		Shandong	*W. pigra*	WP	WP
YP7		Shandong	*W. pigra*	WP	WP
YP5	Stir-frying with rice	Jiangsu	/	/	WP

### Identification of leech species in Chinese patent medicine using 741 F/943R coupled with metabarcoding technology

741 F/943R was used to amplify the barcode regions in seven CPM and a mock community. Illumina sequencing of the amplified products showed that primer 741 F/943R generated 753,883 raw reads, and 508,794 amplicon sequence variant (ASV) reads were obtained after processing with DADA2. These reads were subsequently clustered into 190 ASVs and identified as 13 species (Supplementary Table S3). In the mock community, only five of the introduced leech species were identified. The abundance of reads for *Whitmania pigra*, *Whitmania laevis*, *Hirudo nipponia*, *Poecilobdella manillensis*, and *Erpobdella japonica* was 30.29%, 35.77%, 3.66%, 2.25%, and 28.02% respectively (Supplementary Table S4). According to the mixed proportions, reads for each species should account for 20%. However, there is a significant decrease in the proportion of reads for HN and PM. This could be attributed to the fact that HN and PM, being blood-sucking leeches, have powders that include ingested blood. The close resemblance of read proportions for WP, WL, and EJ to the actual composition indicates that 741 F/943R, coupled with metabarcoding technology, possesses the ability to accurately identify leech species in mixtures.

**Fig. 7:**
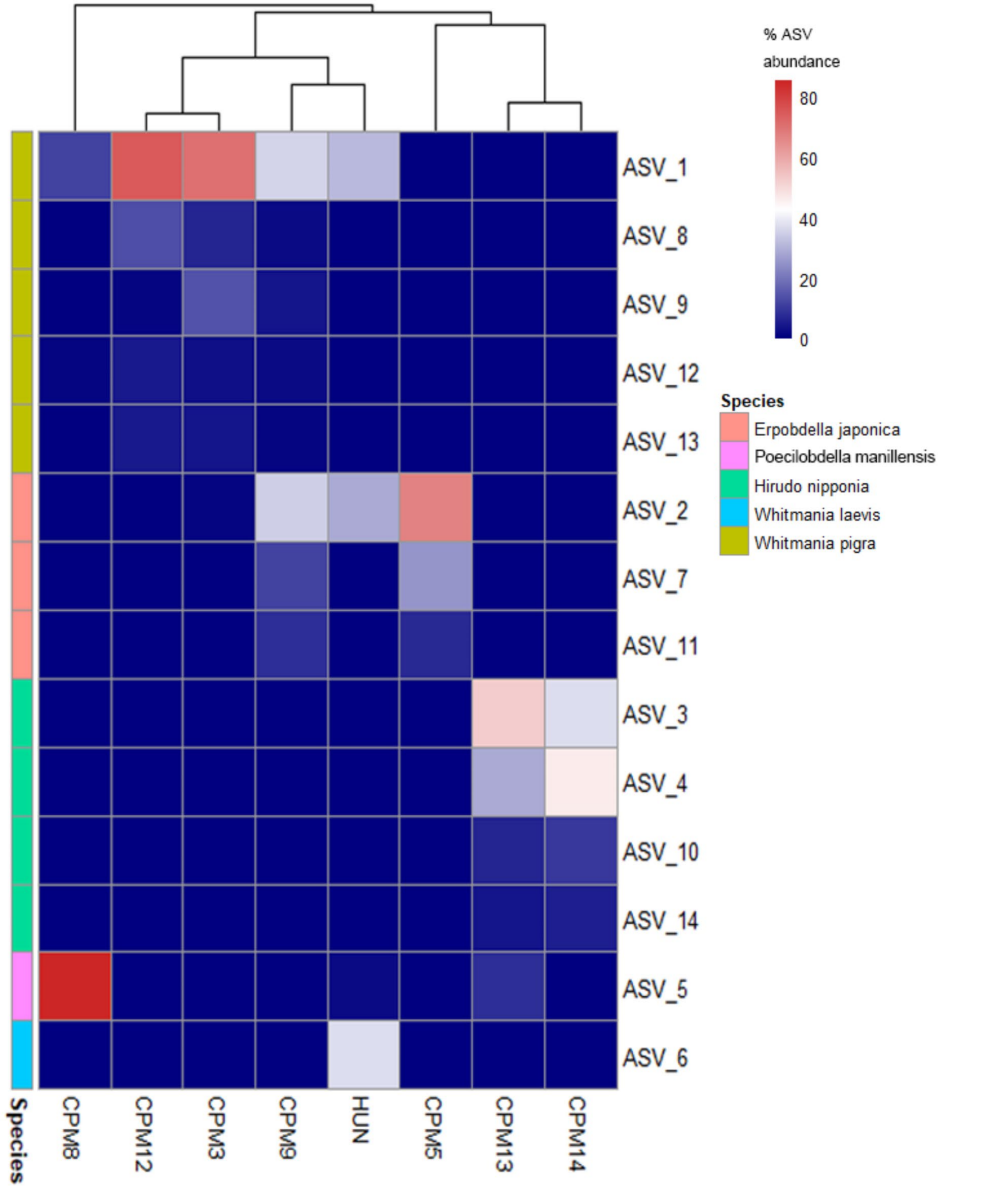
Heat map depicting the distribution of amplicon sequence variant (ASV) read abundance for the primer pair 741 F/943R across experimental mixtures and samples of Chinese patent medicines.

The PCR amplicons of CPM using the 741 F/943R primers were visualized on 1.0% agarose gels, and seven out of fifteen CPM samples were successfully amplified (Supplementary Figure S10). The extraction process of leeches may be a contributing factor to the unsuccessful PCR amplification of other CPM (Supplementary Table S5). Except for CPM8, where 82.44% of reads were annotated, nearly all reads in the remaining CPM received annotations (Supplementary Table S6). CPM8 contains some animal medicines with close phylogenetic relationships to leeches, such as earthworms and scorpions, which may have resulted in non-specific amplification. The identification results and read abundance of leech components in CPM are depicted in Fig. 7, and ASVs with a proportion of reads of less than 1% were excluded. In CPM12, CPM3, and CPM9, the main leech species present are WP, while HN is the predominant species in CPM13 and CPM14. In CPM8, the most abundant leech species is PM, while EJ is also present in CPM12 and CPM5. The results indicate that leech species in CPM are not singular, and even counterfeit species may be present. The mini-barcode of primer 741 F/943R proves effective for identifying leech species in CPM.

## Discussion

Currently, the quality control methods for traditional Chinese medicine primarily involve chemical methodologies, such as thin-layer chromatography, high-performance liquid chromatography, and mass spectrometry^35,36^. However, the chemical components in animal-based medicines are complex, often consisting of steroids, alkaloids, proteins, peptides, and other constituents susceptible to the effects of processing methods and storage conditions^37^. Additionally, chemical component differences sometimes cannot be distinguished between closely related species^38^. Compared with chemical methods, DNA barcoding, due to the stability of DNA against environmental factors, can identify closely related species. Indeed, selecting an appropriate barcode is crucial for the success of DNA barcoding.

An ideal DNA barcode should be easily amplified within the species of interest and possess sufficient variable sites to differentiate closely related species. According to the length, barcodes can be divided into four types: micro-barcode with a length within 100 bp^39^, mini-barcode with a length of 100–250 bp^40^, standard barcode with a length of 400–800 bp^13^, and the whole genome as a super-barcode^41^. Micro-barcode has limited resolution, and although standard barcode and super-barcode have high resolution, they are difficult to amplify in samples where DNA is degraded because of their length. Mini-barcode, with its short length and sufficient variable sites, thus becomes the best choice for the identification of traditional Chinese medicine products. However, high-resolution mini-barcode is not easily found.

*COI* is the most commonly used standard barcode for animal-based medicinal materials^42^. However, our experiments revealed that *COI* did not amplify well in the leech species we collected, especially in WL, where it failed to amplify. Furthermore, HN and PM are blood-feeding leeches, which means that during DNA extraction, host DNA is likely to be extracted as well, potentially leading to sequencing failures, making it difficult to develop a mini-barcode from *COI*. Therefore, we chose to develop mini-barcodes from the mitochondrial genome. After comparing the mitochondrial genomes of five leech species, the *16 S rRNA* gene exhibited greater variability (Fig. 3A) and was easier to design primers (Fig. 4), which led us to choose it for mini-barcode development. Furthermore, 741 F/943R specifically designed for the five leech species avoided the host DNA contamination seen with the universal *COI* primer, LCO1490/HCO2198. Our study also attempted to develop a mini-barcode from *COI.* The results showed that the regions with a Pi value of 0 in the *COI* gene were either too close or too far apart, making it challenging to design primers that would reliably amplify a ~ 200 bp product across all five leech species (Supplementary Figure S11).

Confirming the consistency of the medicinal materials and formulas used in CPM is a challenging task. The processes and formulations of CPM are complex, and existing quality control methods typically rely on chemical means to detect one or several components in CPM preparations, thereby determining the quality of the pharmaceutical products^43,44^. However, this method is not effective in detecting adulteration in CPM. The combination of specific mini-barcodes with metabarcoding technology can facilitate the identification of closely related species^45,46^. In our study, the application of the 741 F/943R primer pair in conjunction with metabarcoding technology successfully detected five species of leeches in a mock community and was able to identify the species of leeches contained in CPM. However, the proportion of reads did not match the mixed proportion, especially for HN and PM. This discrepancy may be due to the fact that they are blood-feeding leeches, and the host’s blood can result in an impure DNA template, making accurate quantification difficult.

Although the universality of our marker has not been sufficiently tested, it can address the identification issue of leech-origin samples. We believe that this mini-barcode method will serve as a valuable guide for quality control research on other herbal medicines and will be continually applied in relevant research fields.

## Methods

### Retrieval and comparative analysis of mitochondrial genome

A total of 5 mitochondrial genomes were downloaded and used from GenBank (https://www.ncbi.nlm.nih.gov/genbank/), including *Hirudo nipponia* (HN), *Poecilobdella manillensis* (PM), *Whitmania acranulata* (WA), *Whitmania laevis* (WL), and *Whitmania pigra* (WP), with the accession numbers NC_023776, NC_023925, NC_023928, NC_023926, and NC_013569, respectively. The physical maps of the five complete mitochondrial genome sequences were visualized with OrganellarGenomeDRAW^47^. The program mVISTA^48^ in Shuffle-LAGAN mode was used to perform the structural comparison of five mitochondrial genomes. At the same time, structural variations between the five leech mitochondrial genomes were further compared by the MAUVE software^49^.

### Identification of hypervariable regions on the mitochondrial genome and design of primers

To find the hypervariable regions, all the genes of five mitochondrial genomes were respectively extracted and aligned using PhyloSuite^50^ v1.2.3. The nucleotide diversity (Pi) values of each gene were calculated using DNAsp^51^ software version 6.12.03. Choosing regions with high Pi values as candidate areas for the development of mini-barcodes. In order to make the primers applicable to various leech species, we performed a sliding window analysis to find conservative regions that have Pi values with 0. The window length was set to 20 bp, and the step size was one bp. Subsequently, Oligo 7^52^ software was used to evaluate the designed primers for physical properties such as annealing temperature, hairpin structures, and primer dimers. Primers with potential hairpin structures, primer dimers, or excessively high annealing temperatures are discarded.

### Evaluation of the efficiency of the mini-barcode primer 741 F/943R for the identification of both fresh and processed leeches

A total of 77 fresh leeches and 70 medicinal leech samples were collected from diverse geographical areas. These samples were classified based on morphological identification, including WP, HN, PM, WL, and *Erpobdella* sp. (Er). TIANamp Feedstuff Animal DNA Kit (Tiangen Biotech, Beijing, China) was used to extract the total genomic DNA. *COI* is a commonly used DNA barcode in animal identification. Therefore, we amplified the *COI* segment of the samples using LCO1490/HCO2198 and the mini-barcode region using 741 F/943R, comparing the identification efficiency of the two methods. PCR amplification was conducted in a 25 µl reaction composed of 12.5 µl of 2 × Taq PCR Mix (Tiangen Biotech, Beijing, China), 1.0 µl of each primer (synthesized by GENEWIZ, Suzhou, China), 1 µl of DNA template, and approximately 9.5 µl ddH2O. The PCR protocol was as follows: preheating at 94 °C for 7 min, 30 cycles at 94 °C for 30 s, annealing at 46.9 °C for 30 s (for LCO1490/HCO2198, the annealing temperature is set to 46.0 °C), and elongation at 72 °C for 1 min, followed by a final extension at 72 °C for 10 min. Negative controls were included in each run. PCR amplicons were visualized on 1.0% agarose gels and then subjected to Sanger sequencing using the same set of primers as for PCR amplification. The sequencing was performed by GENEWIZ on an ABI 3730xl instrument (Applied Biosystems, USA). Proofreading and contig assembly of the sequencing peak diagrams were performed using the CodonCode Aligner (CodonCode, Centerville, MA, USA). The BLAST was carried out using the command line BLAST + executables (version 2.14.1)^53^. A BLAST database was created with 3668 mitochondrial genes of *Hirudinida*. In order to construct the phylogenetic tree, sequences were aligned with MAFFT v7.520^54^ using the “L-INS-i (accurate)” strategy and normal alignment mode. And Gap sites were removed with trimAl v1.2rev57^55^ using the “-automated1” command. Maximum likelihood phylogenies were inferred using IQ-TREE v2.2.5^56^ under the model automatically selected by IQ-TREE (“Auto” option in IQ-TREE) for 1000 Ultrafast bootstraps.

Genomic DNA was extracted from seven batches of leech herbal decoction pieces. Amplification was carried out using LCO1490/HCO2198 and 741 F/943R primers, followed by sequencing. The obtained sequences were then identified for species through BLAST analysis.

### Evaluation of the efficiency of the mini-barcode 741 F/943R in identifying leech species in Chinese patent medicine by metabarcoding

To verify the distinguishing ability of the mini-barcode in complex preparations, a mock community was prepared (Voucher No. HUN) containing 4.85 mg WP, 4.46 mg HN, 6.20 mg HM, 5.27 mg WL, and 5.00 mg Er. Fifteen kinds of CPM containing leech material were purchased from the market. Genomic DNA was extracted from the mock community and each CPM, respectively. The target regions were amplified using fusion primers with matching tags (Supplementary Table S7). The reaction was set up as follows: Genomic DNA (10 ng/µl) 2 µl; amplicon PCR forward primer (10 µM) 1 µl; amplicon PCR reverse primer (10 µM) 1 µl; 2×Hieff^®^ Robust PCR Master Mix (Yeasen, 10105ES03, China) (total 30 µl).

The plate was sealed, and PCR was performed in a thermal instrument (Applied Biosystems 9700, USA) using the following program: 1 cycle of denaturing at 94 °C for 3 min, first 5 cycles of denaturing at 94 °C for 30 s, annealing at 45 °C for 30 s, elongation at 65 °C for 30 s, then 20 cycles of denaturing at 94 °C for 30 s, annealing at 55 °C for 20 s, elongation at 72 °C for 30 s and a final extension at 72 °C for 5 min. The PCR products were checked using electrophoresis in 2% (w/v) agarose gels in TBE buffer (Tris, boric acid, EDTA) stained with ethidium bromide (EB) and visualized under UV light. PCR products were sequenced (2 × 250 bp paired-ends) on the Illumina Miseq system by Sangon Biotech.

The fastq-multx^57^ was used to split data according to the unique tags. The adapter was removed using cutadapt^58^, and primer sequences were trimmed using bbduk^59^. To construct amplicon sequence variants (ASVs), quality control, joining of paired-end reads, removal of chimeras, and denoising were performed with the DADA2^60^. Meanwhile, reads were truncated to exclude low-quality data (N220 bp for forward reads and N190 bp for reverse reads, truncQ = 2, maxEE = 2).

## Electronic supplementary material

Below is the link to the electronic supplementary material.

Supplementary Material 1

## Data Availability

The datasets generated and during the current study are available in the GenBank: PP955589-PP955690 and https://www.ncbi.nlm.nih.gov/nuccore/PP955589 for COX1, PP960368-PP960511 and https://www.ncbi.nlm.nih.gov/nuccore/PP960368 for self-designed mini-barcode. The raw sequence data of metabarcoding for this study also can be found in GenBank. The associated BioProject is PRJNA1128649, https://www.ncbi.nlm.nih.gov/bioproject/1128649.
