# Supplementary material for: Development of a mitochondrial mini-barcode and its application in metabarcoding for identification of leech in traditional Chinese medicine
Source: Sci Rep. 2025 Jan 11;15:1698. doi: 10.1038/s41598-024-77913-6 (PMC11724943; doi:10.1038/s41598-024-77913-6)
Supplement: Supplementary file 1 — Supplementary Material 1 [file 41598_2024_77913_MOESM1_ESM.docx]

Supporting Information for

**Advancing Species Identification in Traditional Chinese Medicine: A Novel Mini-Barcode from Mitochondrial Genomes Combined with Meta-barcoding Technology for Leech Authentication**

Chenfeng Shi^1^, Yuhan Guo^1^, Lijuan Yao^1^, Yunhui Xu^1^, Jing Zhou^1^, and Moli Hua^1, *^

^1^ National Key Laboratory of Lead Druggability Research, Shanghai Institute of pharmaceutical industry, China State Institute of Pharmaceutical Industry, Shanghai 201203, People’s Republic of China
^*^huamoli@sinopharm.com


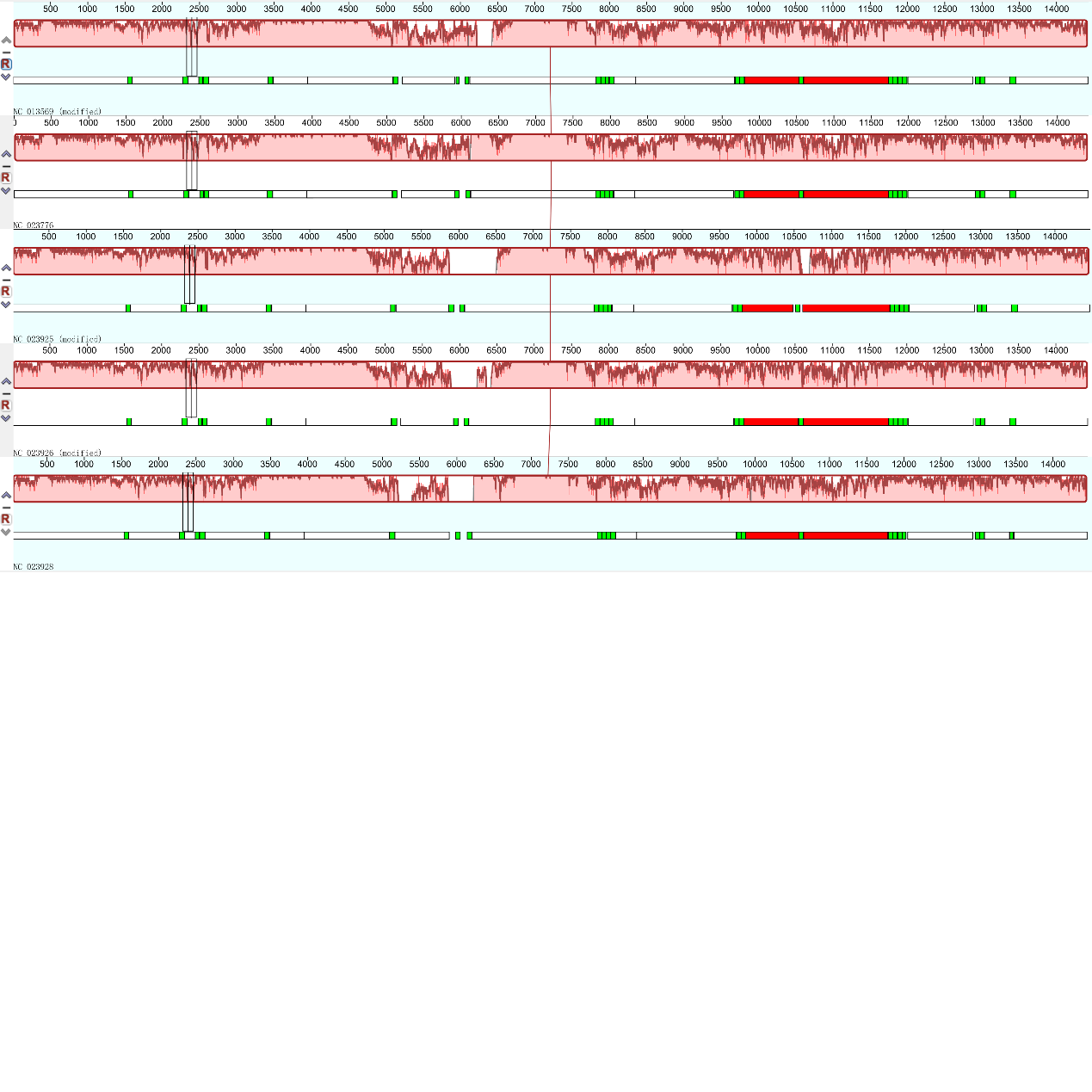


Figure S1 The result of the mitochondrial genome structure comparison using MAUVE.


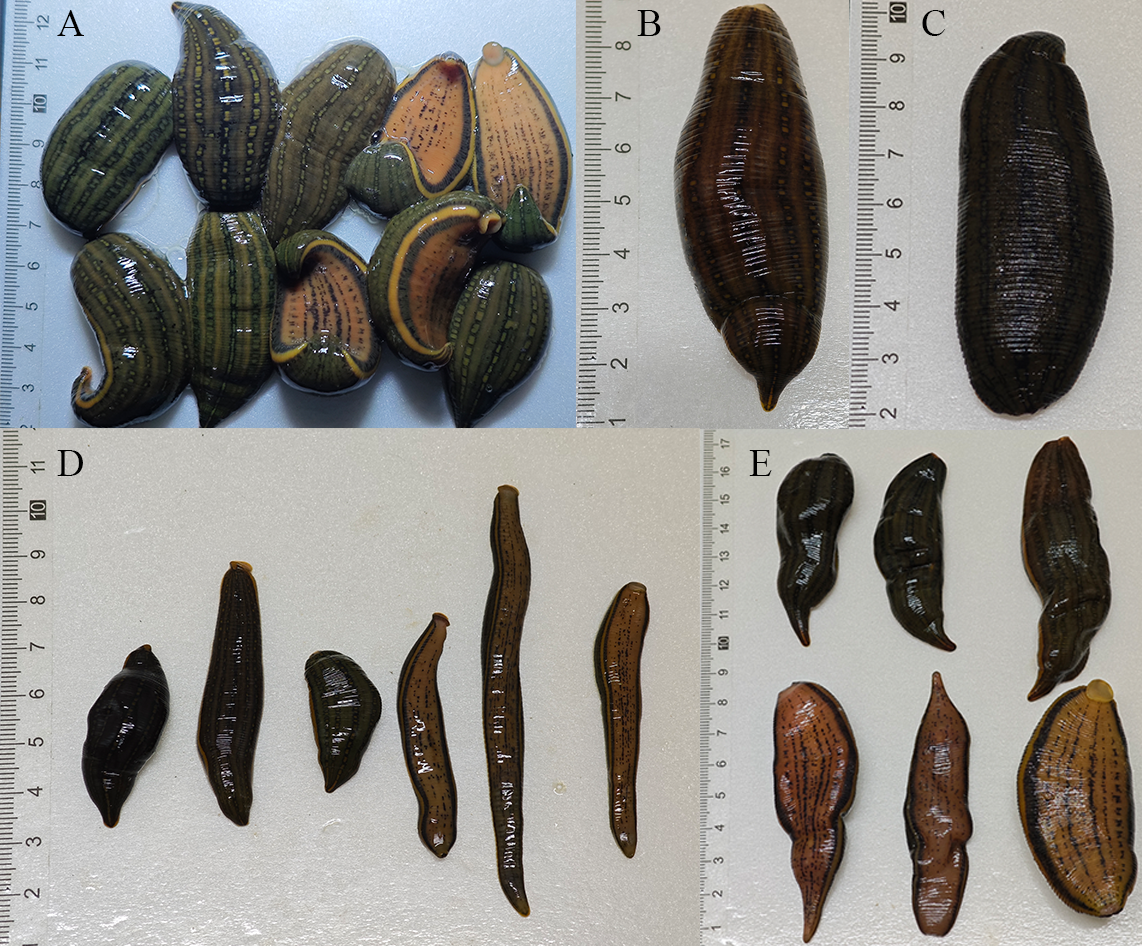


Figure S2 Fresh samples of *Whitmania pigra*. A: WPZJ01-10; B: WPJX01; C: WPSH01; D: WPSD01-06; E: WPJS01-06.


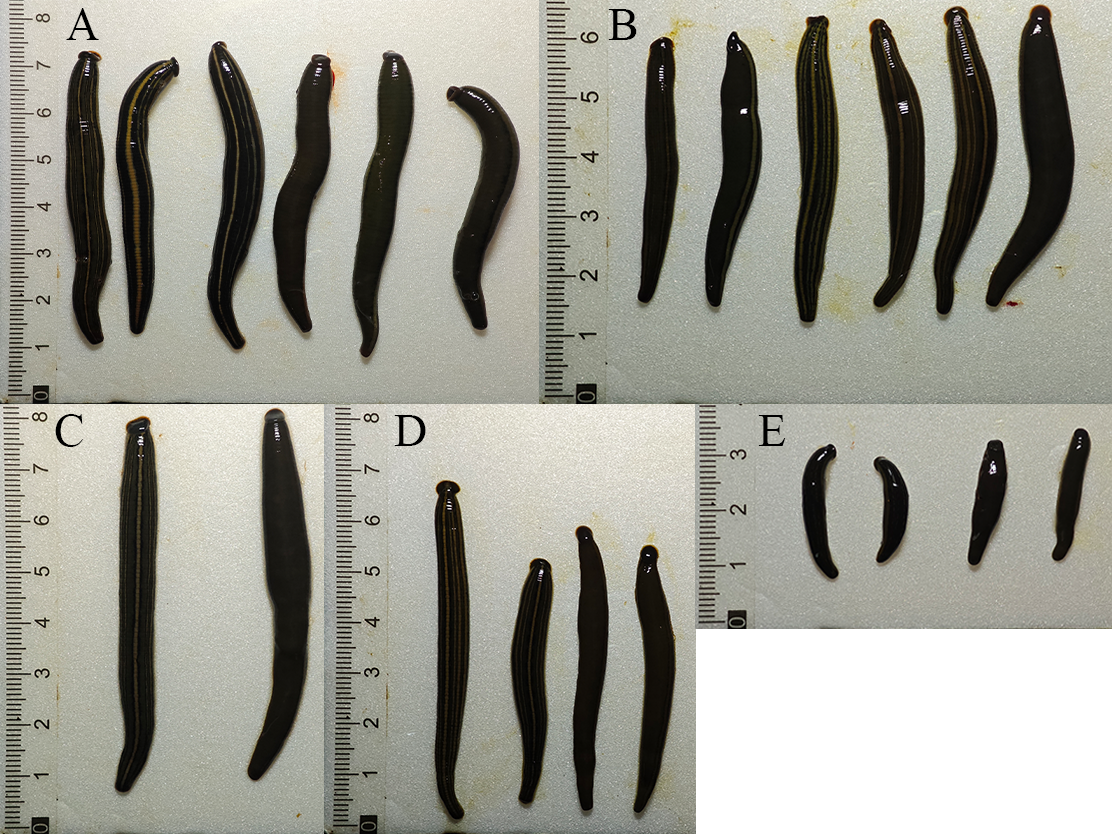


Figure S3 Fresh samples of *Hirudo nipponia*. A: HNGD01-06; B: HNSD01-06; C: HNJZ01-02; D: HNHB01-04; E: HNTJ01-04.


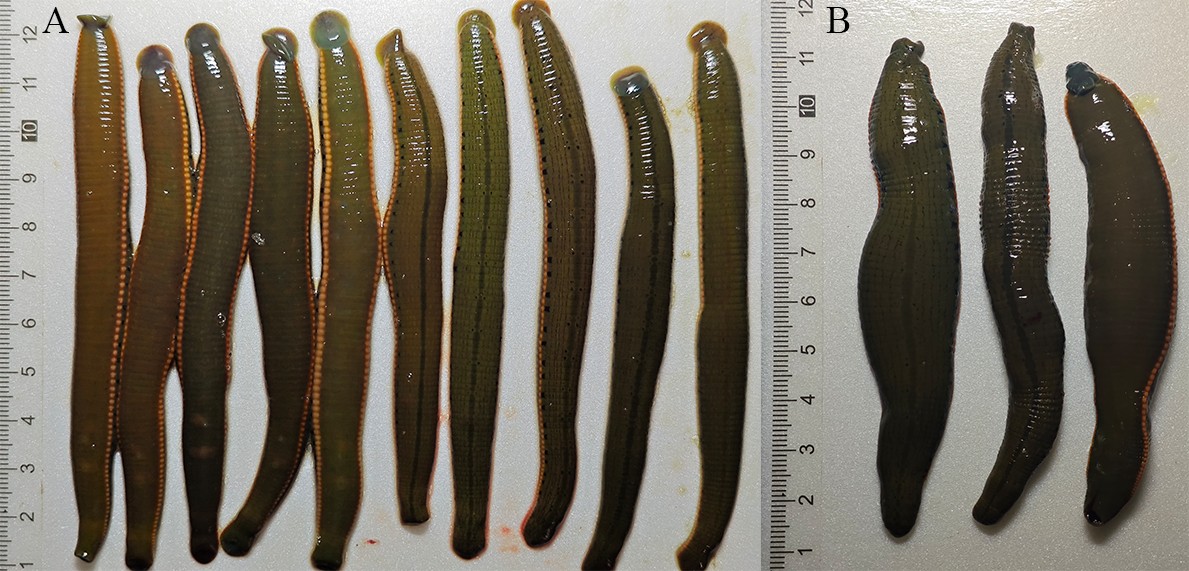


Figure S4 Fresh samples of *Poecilobdella manillensis*. A: PMYN01-10; B: PMGD01-03.


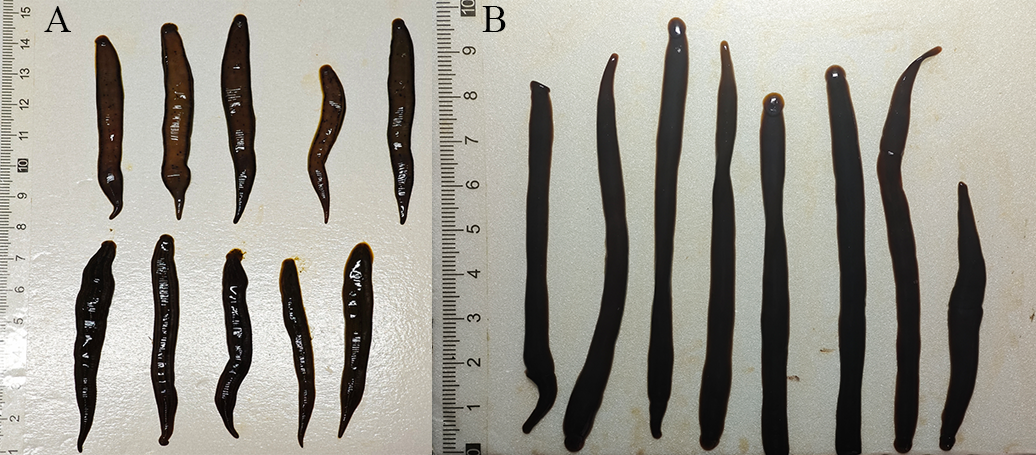


Figure S5 Fresh samples of *Whitmania laevis*, WLSH01-10 (A) and *Erpobdella* sp., ErSY01-08 (B).


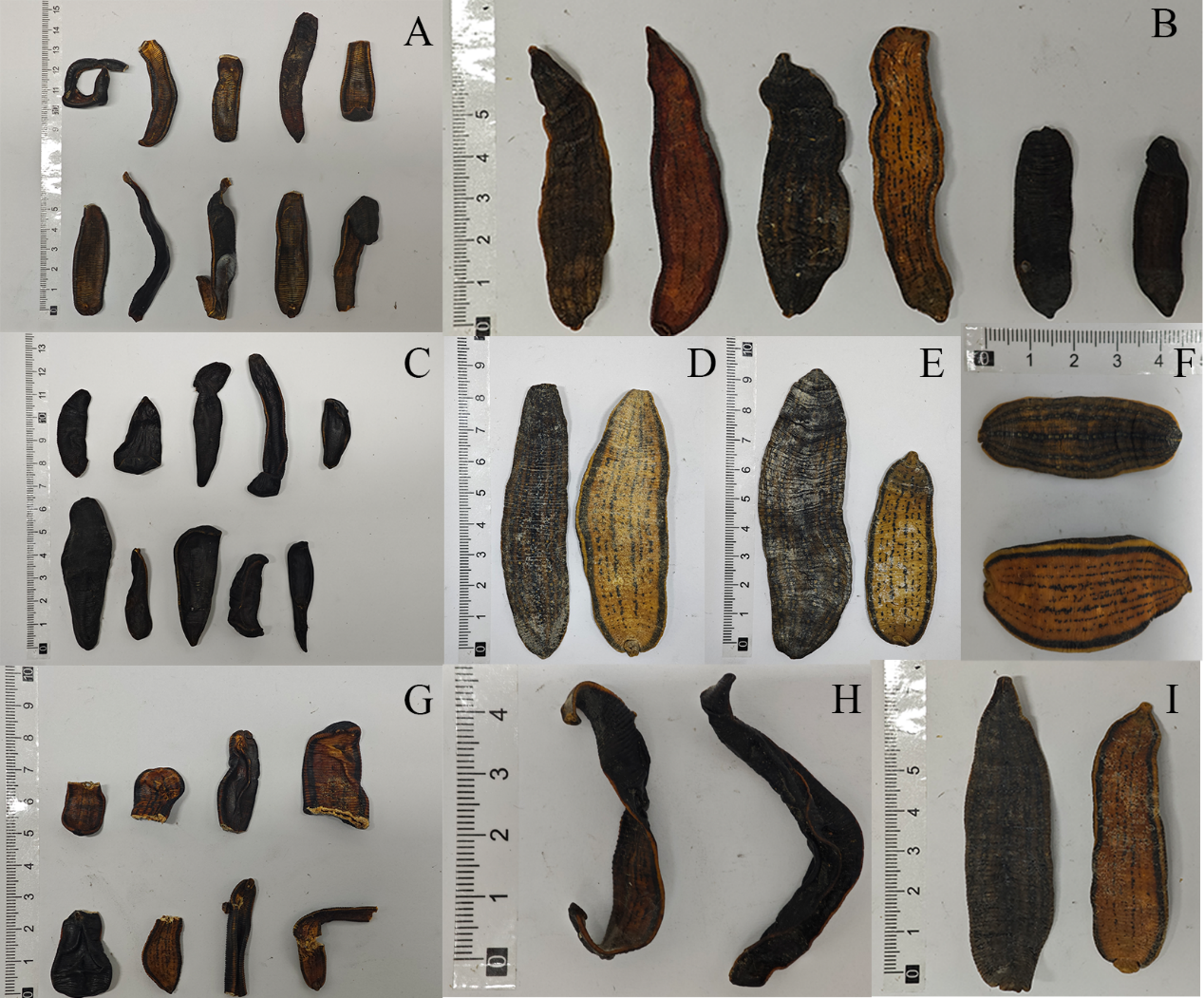


Figure S6 Medicinal material of *Whitmania pigra*. A, C, D, E and G: WPAH01-32; B: WPJS01-06; F: WPHLJ01-02; H: WPLN01-02; I: WPHN01-02.


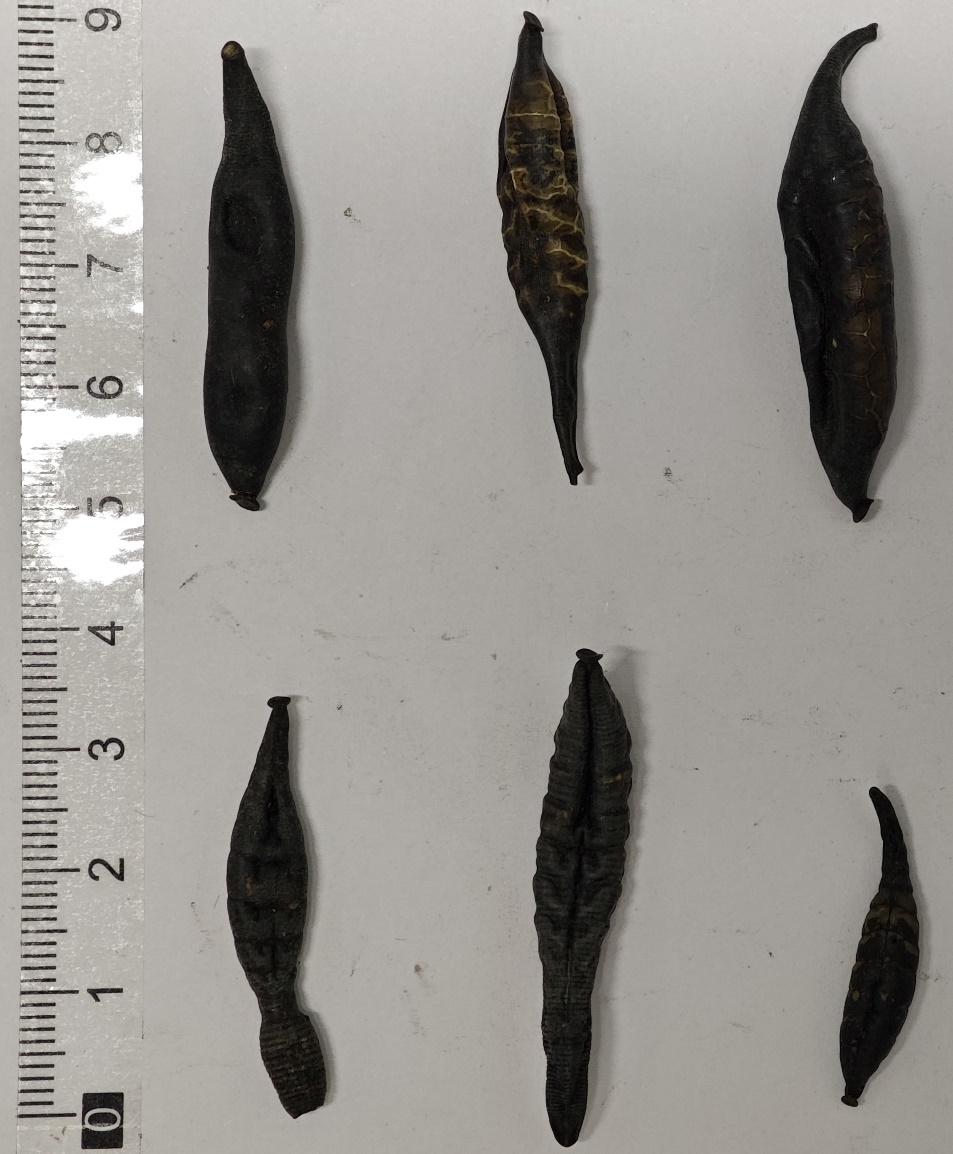


Figure S7 Medicinal material of *Hirudo nipponia*. HNAH01-06


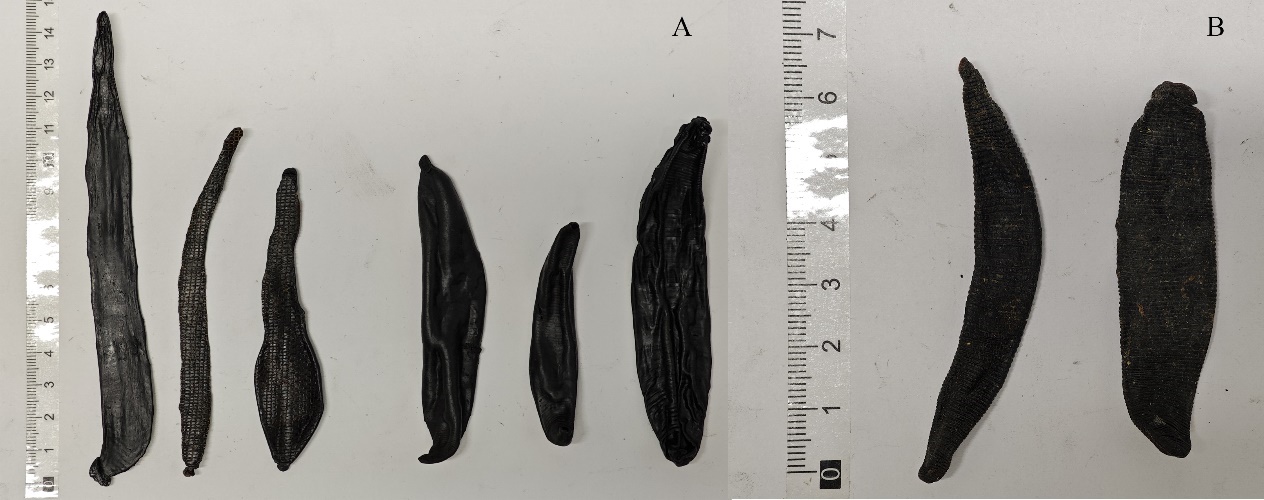


Figure S8 Medicinal material of *Poecilobdella manillensis*. A. PMAH01-06; B. PMMyanmar01-02.


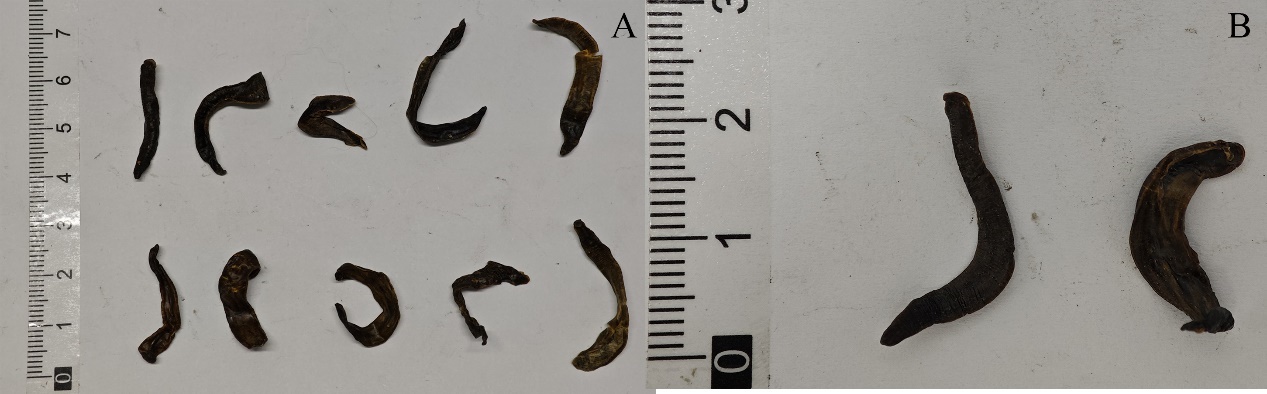


Figure S9 Medicinal material of *Erpobdella* sp. A. ErAH01-10; B. EJLN01-02.

\


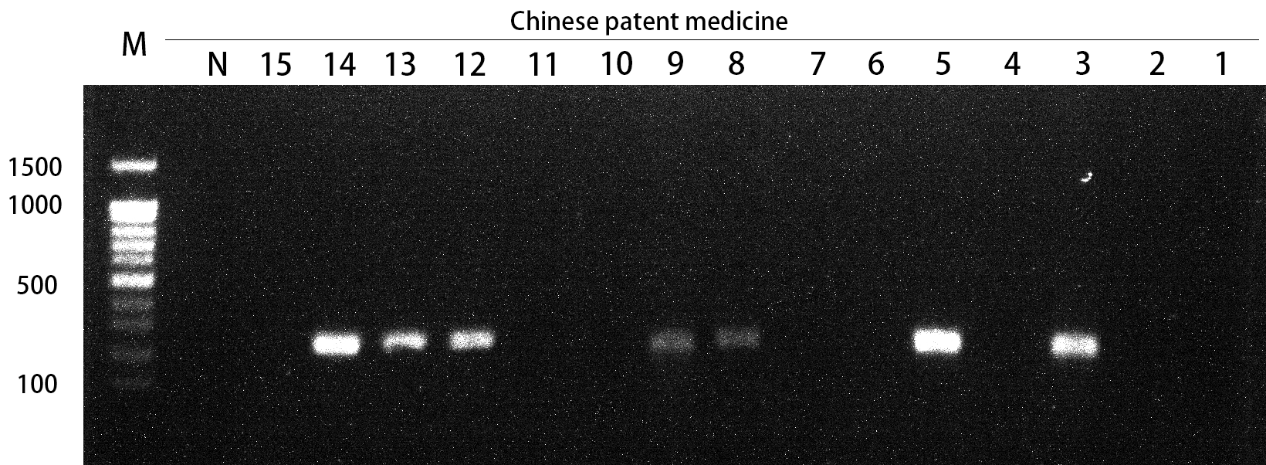


Figure S10 The gel electrophoresis results of the amplification of DNA barcodes using designed mini-barcode primer 741F/943R. Lane M was the marker. The lanes from left to right corresponded to products from CPM 1-15.


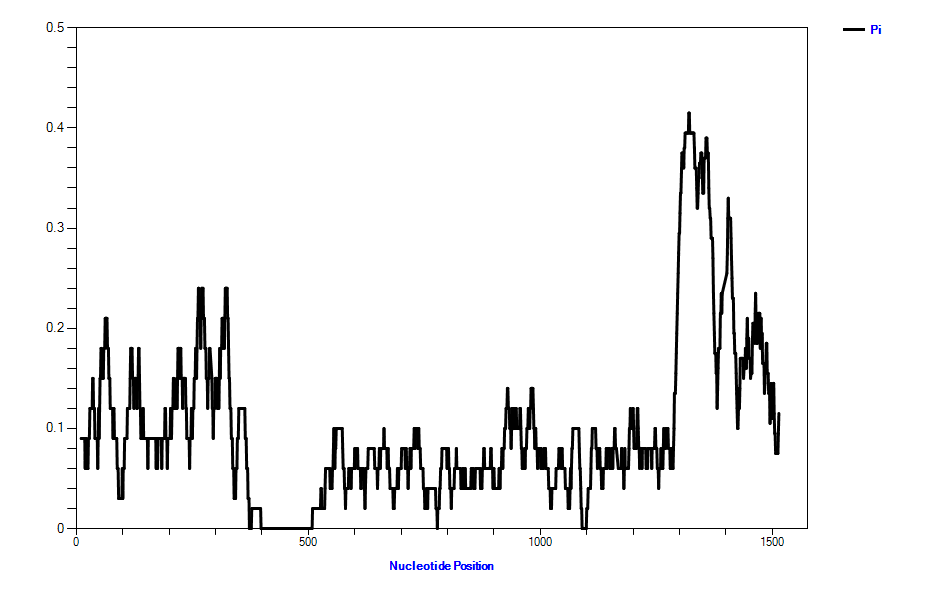


Figure S11 Sliding window analysis of nucleotide variability in the *COI* gene.

Supplementary Table S1 BLAST results of *COI* fragment for the samples.

| qaccver | saccver | sscinames | pident | evalue | length | mismatch |
| --- | --- | --- | --- | --- | --- | --- |
| EJAH01 | MF358688.1 | Erpobdella japonica | 98.831 | 0 | 599 | 7 |
| EJAH02 | KM095091.1 | Erpobdella sp. IAK-2015a | 98.331 | 0 | 599 | 10 |
| EJAH03 | MF358688.1 | Erpobdella japonica | 98.164 | 0 | 599 | 11 |
| EJAH04 | KM095091.1 | Erpobdella sp. IAK-2015a | 97.997 | 0 | 599 | 12 |
| EJAH05 | KM095091.1 | Erpobdella sp. IAK-2015a | 98.164 | 0 | 599 | 11 |
| EJAH06 | KM095091.1 | Erpobdella sp. IAK-2015a | 97.997 | 0 | 599 | 12 |
| EJAH07 | KM095091.1 | Erpobdella sp. IAK-2015a | 98.164 | 0 | 599 | 11 |
| EJAH08 | KM095091.1 | Erpobdella sp. IAK-2015a | 98.998 | 0 | 599 | 6 |
| EJAH09 | KM095091.1 | Erpobdella sp. IAK-2015a | 97.997 | 0 | 599 | 12 |
| EJAH10 | KM095091.1 | Erpobdella sp. IAK-2015a | 97.496 | 0 | 599 | 15 |
| EJLN01 | MF358688.1 | Erpobdella japonica | 98.497 | 0 | 599 | 9 |
| EJLN02 | MF358688.1 | Erpobdella japonica | 97.997 | 0 | 599 | 12 |
| EJSY07 | KM095091.1 | Erpobdella sp. IAK-2015a | 97.83 | 0 | 599 | 13 |
| EJSY08 | KM095091.1 | Erpobdella sp. IAK-2015a | 97.997 | 0 | 599 | 12 |
| HNAH01 | CM079106.1 | Hirudo tianjinensis | 100 | 0 | 599 | 0 |
| HNAH02 | OL824780.1 | Hirudo nipponia | 100 | 0 | 599 | 0 |
| HNAH03 | CM079106.1 | Hirudo tianjinensis | 100 | 0 | 599 | 0 |
| HNAH04 | OR578844.1 | Hirudo nipponia | 100 | 0 | 599 | 0 |
| HNAH05 | CM079106.1 | Hirudo tianjinensis | 99.666 | 0 | 599 | 2 |
| HNAH06 | OL824777.1 | Hirudo nipponia | 100 | 0 | 599 | 0 |
| HNGD01 | CM079106.1 | Hirudo tianjinensis | 99.662 | 0 | 591 | 2 |
| HNGD02 | CM079106.1 | Hirudo tianjinensis | 100 | 0 | 555 | 0 |
| HNGD03 | CM079106.1 | Hirudo tianjinensis | 99.499 | 0 | 599 | 3 |
| HNGD04 | CM079106.1 | Hirudo tianjinensis | 100 | 0 | 599 | 0 |
| HNGD05 | CM079106.1 | Hirudo tianjinensis | 99.832 | 0 | 595 | 1 |
| HNGD06 | CM079106.1 | Hirudo tianjinensis | 99.832 | 0 | 597 | 1 |
| HNHB01 | MZ820661.1 | Hirudo nipponia | 100 | 0 | 599 | 0 |
| HNHB02 | MZ820661.1 | Hirudo nipponia | 100 | 0 | 599 | 0 |
| HNHB03 | MZ820661.1 | Hirudo nipponia | 99.833 | 0 | 599 | 1 |
| HNJZ01 | CM079106.1 | Hirudo tianjinensis | 97.158 | 0 | 563 | 16 |
| HNJZ02 | OL824776.1 | Hirudo nipponia | 98.993 | 0 | 596 | 6 |
| HNSD01 | CM079106.1 | Hirudo tianjinensis | 99.828 | 0 | 581 | 1 |
| HNSD02 | OR578876.1 | Hirudo nipponia | 100 | 0 | 578 | 0 |
| HNSD03 | CM079106.1 | Hirudo tianjinensis | 100 | 0 | 599 | 0 |
| HNSD04 | CM079106.1 | Hirudo tianjinensis | 100 | 0 | 599 | 0 |
| HNSD05 | CM079106.1 | Hirudo tianjinensis | 100 | 0 | 599 | 0 |
| HNTJ01 | MZ820657.1 | Hirudo tianjinensis | 99.833 | 0 | 599 | 1 |
| HNTJ02 | MZ820656.1 | Hirudo tianjinensis | 99.833 | 0 | 599 | 1 |
| HNTJ03 | MZ820661.1 | Hirudo nipponia | 100 | 0 | 599 | 0 |
| HNTJ04 | MZ820661.1 | Hirudo nipponia | 99.833 | 0 | 599 | 1 |
| PMAH01 | MK111109.1 | Hirudinaria sp. XX-2018 | 100 | 0 | 599 | 0 |
| PMAH02 | MK111109.1 | Hirudinaria sp. XX-2018 | 100 | 0 | 599 | 0 |
| PMAH03 | MK111109.1 | Hirudinaria sp. XX-2018 | 100 | 0 | 599 | 0 |
| PMGD02 | OR578880.1 | Poecilobdella manillensis | 99.833 | 0 | 599 | 1 |
| PMGD03 | MK111109.1 | Hirudinaria sp. XX-2018 | 99.812 | 0 | 531 | 1 |
| PMMyanmar02 | KU870761.1 | Poecilobdella manillensis | 98.325 | 0 | 597 | 10 |
| PMSD01 | MK111109.1 | Hirudinaria sp. XX-2018 | 100 | 0 | 599 | 0 |
| PMSD02 | MK111109.1 | Hirudinaria sp. XX-2018 | 100 | 0 | 599 | 0 |
| PMSD08 | MK111109.1 | Hirudinaria sp. XX-2018 | 100 | 0 | 599 | 0 |
| PMSD09 | OR578880.1 | Poecilobdella manillensis | 99.833 | 0 | 599 | 1 |
| PMSD10 | OR578880.1 | Poecilobdella manillensis | 99.833 | 0 | 599 | 1 |
| WPAH01 | KC688268.1 | Poecilobdella manillensis | 100 | 0 | 599 | 0 |
| WPAH04 | KC688268.1 | Poecilobdella manillensis | 100 | 0 | 599 | 0 |
| WPAH05 | OR578861.1 | Whitmania pigra | 99.666 | 0 | 599 | 2 |
| WPAH06 | MW659834.1 | Whitmania pigra | 99.833 | 0 | 599 | 1 |
| WPAH07 | KC688268.1 | Poecilobdella manillensis | 100 | 0 | 599 | 0 |
| WPAH08 | OR578858.1 | Whitmania pigra | 99.833 | 0 | 599 | 1 |
| WPAH10 | EU304459.1 | Whitmania pigra | 100 | 0 | 599 | 0 |
| WPAH11 | OR578861.1 | Whitmania pigra | 99.666 | 0 | 599 | 2 |
| WPAH13 | OR578858.1 | Whitmania pigra | 100 | 0 | 599 | 0 |
| WPAH14 | OR578860.1 | Whitmania pigra | 100 | 0 | 599 | 0 |
| WPAH15 | OR578861.1 | Whitmania pigra | 99.666 | 0 | 599 | 2 |
| WPAH16 | MW659834.1 | Whitmania pigra | 99.833 | 0 | 599 | 1 |
| WPAH17 | OR578860.1 | Whitmania pigra | 99.666 | 0 | 599 | 2 |
| WPAH18 | OR578861.1 | Whitmania pigra | 98.998 | 0 | 599 | 6 |
| WPAH19 | OR578859.1 | Whitmania pigra | 100 | 0 | 599 | 0 |
| WPAH20 | OR578859.1 | Whitmania pigra | 100 | 0 | 599 | 0 |
| WPAH21 | MW659834.1 | Whitmania pigra | 99.833 | 0 | 599 | 1 |
| WPAH23 | KC688268.1 | Poecilobdella manillensis | 100 | 0 | 599 | 0 |
| WPAH24 | EU304459.1 | Whitmania pigra | 100 | 0 | 599 | 0 |
| WPAH25 | OR578860.1 | Whitmania pigra | 99.666 | 0 | 599 | 2 |
| WPAH26 | OR578861.1 | Whitmania pigra | 98.831 | 0 | 599 | 7 |
| WPAH27 | OR578858.1 | Whitmania pigra | 100 | 0 | 599 | 0 |
| WPAH28 | OR578861.1 | Whitmania pigra | 99.332 | 0 | 599 | 4 |
| WPAH29 | OR578861.1 | Whitmania pigra | 99.833 | 0 | 599 | 1 |
| WPAH31 | KC688268.1 | Poecilobdella manillensis | 100 | 0 | 599 | 0 |
| WPDL01 | OR578858.1 | Whitmania pigra | 100 | 0 | 599 | 0 |
| WPDL02 | OR578858.1 | Whitmania pigra | 100 | 0 | 599 | 0 |
| WPHN02 | KU553102.1 | Barbronia weberi | 8.47E+01 | 3.19E-170 | 593 | 91 |
| WPZJ09 | MZ820661.1 | Hirudo nipponia | 99.833 | 0 | 599 | 1 |
| WPZJ10 | MW659834.1 | Whitmania pigra | 99.666 | 0 | 599 | 2 |
| WPJS01 | OR578857.1 | Whitmania pigra | 99.833 | 0 | 599 | 1 |
| WPJS02 | OR578860.1 | Whitmania pigra | 100 | 0 | 599 | 0 |
| WPJS06 | OR578861.1 | Whitmania pigra | 99.666 | 0 | 599 | 2 |
| WPJX | OR578861.1 | Whitmania pigra | 99.499 | 0 | 599 | 3 |
| WPKS01 | OR578859.1 | Whitmania pigra | 100 | 0 | 599 | 0 |
| WPKS02 | OR578861.1 | Whitmania pigra | 98.664 | 0 | 599 | 8 |
| WPKS03 | OR578859.1 | Whitmania pigra | 100 | 0 | 599 | 0 |
| WPKS04 | OR578861.1 | Whitmania pigra | 98.497 | 0 | 599 | 9 |
| WPKS06 | OR578857.1 | Whitmania pigra | 99.833 | 0 | 599 | 1 |
| WPSD01 | OR578858.1 | Whitmania pigra | 99.499 | 0 | 599 | 3 |
| WPSD02 | KC688268.1 | Poecilobdella manillensis | 100 | 0 | 599 | 0 |
| WPSD03 | KC688268.1 | Poecilobdella manillensis | 100 | 0 | 599 | 0 |
| WPSD04 | KC688268.1 | Poecilobdella manillensis | 100 | 0 | 599 | 0 |
| WPSD05 | KC688268.1 | Poecilobdella manillensis | 100 | 0 | 599 | 0 |
| WPSD06 | KC688268.1 | Poecilobdella manillensis | 99.833 | 0 | 599 | 1 |
| WPZJ01 | OR578861.1 | Whitmania pigra | 98.998 | 0 | 599 | 6 |
| WPZJ04 | OQ076768.1 | Whitmania pigra | 97.97 | 0 | 542 | 11 |
| WPZJ05 | OR578861.1 | Whitmania pigra | 98.497 | 0 | 599 | 9 |
| WPZJ06 | OR578861.1 | Whitmania pigra | 99.666 | 0 | 599 | 2 |
| WPZJ07 | OR578861.1 | Whitmania pigra | 97.292 | 0 | 517 | 14 |
| WPZJ08 | OQ076768.1 | Whitmania pigra | 97.007 | 0 | 568 | 17 |
| YP3 | MF358688.1 | Erpobdella japonica | 98.831 | 0 | 599 | 7 |
| YP4 | KM095091.1 | Erpobdella sp. IAK-2015a | 98.331 | 0 | 599 | 10 |
| YP6 | MF358688.1 | Erpobdella japonica | 98.164 | 0 | 599 | 11 |
| YP7 | KM095091.1 | Erpobdella sp. IAK-2015a | 97.997 | 0 | 599 | 12 |

Supplementary Table S2 BLAST result of 741F/943R for the samples.

| qaccver | saccver | sscinames | pident | evalue | length | mismatch |
| --- | --- | --- | --- | --- | --- | --- |
| EJAH01 | MF358688.1 | Erpobdella japonica | 99.07 | 1.99E-108 | 215 | 2 |
| EJAH02 | MF358688.1 | Erpobdella japonica | 99.479 | 2.31E-97 | 192 | 1 |
| EJAH03 | MF358688.1 | Erpobdella japonica | 99.057 | 9.10E-107 | 212 | 2 |
| EJAH04 | MF358688.1 | Erpobdella japonica | 99.057 | 9.10E-107 | 212 | 2 |
| EJAH05 | MF358688.1 | Erpobdella japonica | 99.074 | 5.55E-109 | 216 | 2 |
| EJAH06 | MF358688.1 | Erpobdella japonica | 99.065 | 7.14E-108 | 214 | 2 |
| EJAH07 | CM079106.1 | Hirudo tianjinensis | 99.541 | 9.74E-112 | 218 | 1 |
| EJAH08 | CM079106.1 | Hirudo tianjinensis | 100 | 1.63E-114 | 220 | 0 |
| EJAH09 | CM079106.1 | Hirudo tianjinensis | 99.548 | 7.38E-113 | 221 | 0 |
| EJAH10 | KC667144.1 | Hirudo nipponia | 99.548 | 7.46E-113 | 221 | 0 |
| EJLN01 | CM079106.1 | Hirudo tianjinensis | 99.095 | 3.60E-111 | 221 | 1 |
| EJLN02 | KC667144.1 | Hirudo nipponia | 99.087 | 1.27E-110 | 219 | 2 |
| EJSY01 | CM079106.1 | Hirudo tianjinensis | 99.552 | 1.60E-114 | 223 | 1 |
| EJSY02 | CM079106.1 | Hirudo tianjinensis | 99.548 | 7.49E-113 | 221 | 0 |
| EJSY03 | CM079106.1 | Hirudo tianjinensis | 99.548 | 7.57E-113 | 221 | 0 |
| EJSY04 | CM079106.1 | Hirudo tianjinensis | 100 | 1.24E-115 | 222 | 0 |
| EJSY05 | CM079106.1 | Hirudo tianjinensis | 100 | 1.61E-114 | 220 | 0 |
| EJSY06 | CM079106.1 | Hirudo tianjinensis | 100 | 4.50E-115 | 221 | 0 |
| EJSY07 | MZ507570.1 | Hirudo nipponia | 100 | 1.23E-115 | 222 | 0 |
| EJSY08 | MZ507570.1 | Hirudo nipponia | 99.556 | 1.26E-115 | 225 | 1 |
| HNAH01 | MZ507570.1 | Hirudo nipponia | 100 | 1.59E-114 | 220 | 0 |
| HNAH02 | MZ507570.1 | Hirudo nipponia | 99.554 | 4.59E-115 | 224 | 1 |
| HNAH03 | KC667144.1 | Hirudo nipponia | 98.19 | 2.16E-108 | 221 | 4 |
| HNAH04 | KC667144.1 | Hirudo nipponia | 99.095 | 3.47E-111 | 221 | 1 |
| HNAH05 | CM079106.1 | Hirudo tianjinensis | 99.095 | 3.47E-111 | 221 | 1 |
| HNAH06 | CM079106.1 | Hirudo tianjinensis | 99.543 | 9.88E-112 | 219 | 0 |
| HNGD01 | CM079106.1 | Hirudo tianjinensis | 97.738 | 3.58E-106 | 221 | 3 |
| HNGD02 | CM079106.1 | Hirudo tianjinensis | 99.545 | 2.72E-112 | 220 | 0 |
| HNGD03 | CM079106.1 | Hirudo tianjinensis | 99.548 | 7.57E-113 | 221 | 0 |
| HNGD04 | CM079106.1 | Hirudo tianjinensis | 97.309 | 3.53E-106 | 223 | 4 |
| HNGD05 | CM079106.1 | Hirudo tianjinensis | 99.115 | 5.79E-114 | 226 | 1 |
| HNGD06 | MZ507570.1 | Hirudo nipponia | 100 | 1.59E-114 | 220 | 0 |
| HNHB01 | MZ507570.1 | Hirudo nipponia | 98.222 | 4.62E-110 | 225 | 3 |
| HNHB02 | KC688268.1 | Poecilobdella manillensis | 100 | 1.27E-120 | 231 | 0 |
| HNHB03 | KC688268.1 | Poecilobdella manillensis | 100 | 1.27E-120 | 231 | 0 |
| HNHB04 | KC688268.1 | Poecilobdella manillensis | 100 | 1.30E-120 | 231 | 0 |
| HNJZ01 | KC688268.1 | Poecilobdella manillensis | 98.246 | 2.84E-112 | 228 | 4 |
| HNJZ02 | KC688268.1 | Poecilobdella manillensis | 95.259 | 8.11E-103 | 232 | 11 |
| HNSD01 | KC688268.1 | Poecilobdella manillensis | 100 | 1.30E-120 | 231 | 0 |
| HNSD02 | KC688268.1 | Poecilobdella manillensis | 99.571 | 1.65E-119 | 233 | 0 |
| HNSD03 | KC688268.1 | Poecilobdella manillensis | 100 | 1.30E-120 | 231 | 0 |
| HNSD04 | KC688268.1 | Poecilobdella manillensis | 100 | 1.28E-120 | 231 | 0 |
| HNSD05 | AF315058.1 | Hirudo medicinalis | 96.93 | 2.77E-107 | 228 | 7 |
| HNTJ01 | AF315058.1 | Hirudo medicinalis | 96.943 | 7.77E-108 | 229 | 7 |
| HNTJ02 | KC688268.1 | Poecilobdella manillensis | 100 | 1.28E-120 | 231 | 0 |
| HNTJ03 | KC688268.1 | Poecilobdella manillensis | 100 | 3.59E-121 | 232 | 0 |
| HNTJ04 | KC688268.1 | Poecilobdella manillensis | 100 | 1.29E-120 | 231 | 0 |
| PMAH01 | KC688268.1 | Poecilobdella manillensis | 100 | 1.30E-120 | 231 | 0 |
| PMAH02 | KC688268.1 | Poecilobdella manillensis | 100 | 3.59E-121 | 232 | 0 |
| PMAH03 | KC688268.1 | Poecilobdella manillensis | 99.565 | 2.17E-118 | 230 | 1 |
| PMAH04 | KC688268.1 | Poecilobdella manillensis | 100 | 3.59E-121 | 232 | 0 |
| PMAH05 | KC688268.1 | Poecilobdella manillensis | 100 | 1.28E-120 | 231 | 0 |
| PMAH06 | KC688268.1 | Poecilobdella manillensis | 100 | 1.27E-120 | 231 | 0 |
| PMGD01 | KC688268.1 | Poecilobdella manillensis | 100 | 9.93E-122 | 233 | 0 |
| PMGD02 | KC688269.1 | Whitmania laevis | 98.673 | 2.75E-112 | 226 | 2 |
| PMGD03 | KC688269.1 | Whitmania laevis | 100 | 4.41E-115 | 221 | 0 |
| PMMyanmar01 | KC688269.1 | Whitmania laevis | 100 | 5.68E-114 | 219 | 0 |
| PMMyanmar02 | KC688269.1 | Whitmania laevis | 99.548 | 2.05E-113 | 221 | 1 |
| PMSD01 | KC688269.1 | Whitmania laevis | 100 | 4.41E-115 | 221 | 0 |
| PMSD02 | KC688269.1 | Whitmania laevis | 100 | 5.68E-114 | 219 | 0 |
| PMSD03 | KC688269.1 | Whitmania laevis | 100 | 5.68E-114 | 219 | 0 |
| PMSD04 | KC688269.1 | Whitmania laevis | 99.545 | 2.66E-112 | 220 | 0 |
| PMSD05 | KC688269.1 | Whitmania laevis | 99.103 | 2.69E-112 | 223 | 1 |
| PMSD06 | EU304459.1 | Whitmania pigra | 100 | 1.26E-89 | 175 | 0 |
| PMSD07 | EU304459.1 | Whitmania pigra | 99.554 | 1.62E-114 | 224 | 0 |
| PMSD08 | EU304459.1 | Whitmania pigra | 100 | 5.68E-114 | 219 | 0 |
| PMSD09 | EU304459.1 | Whitmania pigra | 99.552 | 5.79E-114 | 223 | 0 |
| PMSD10 | EU304459.1 | Whitmania pigra | 99.111 | 5.79E-114 | 225 | 2 |
| WLSH01 | EU304459.1 | Whitmania pigra | 99.552 | 5.74E-114 | 223 | 0 |
| WLSH02 | EU304459.1 | Whitmania pigra | 99.545 | 7.35E-113 | 220 | 1 |
| WLSH03 | EU304459.1 | Whitmania pigra | 100 | 5.71E-114 | 219 | 0 |
| WLSH04 | EU304459.1 | Whitmania pigra | 99.548 | 2.05E-113 | 221 | 1 |
| WLSH05 | EU304459.1 | Whitmania pigra | 99.552 | 1.60E-114 | 223 | 1 |
| WLSH06 | EU304459.1 | Whitmania pigra | 100 | 5.68E-114 | 219 | 0 |
| WLSH07 | EU304459.1 | Whitmania pigra | 100 | 5.71E-114 | 219 | 0 |
| WLSH08 | EU304459.1 | Whitmania pigra | 100 | 5.74E-114 | 219 | 0 |
| WLSH09 | EU304459.1 | Whitmania pigra | 99.552 | 5.76E-114 | 223 | 0 |
| WPAH01 | EU304459.1 | Whitmania pigra | 99.103 | 2.69E-112 | 223 | 1 |
| WPAH03 | EU304459.1 | Whitmania pigra | 99.524 | 9.76E-107 | 210 | 0 |
| WPAH04 | EU304459.1 | Whitmania pigra | 100 | 7.53E-113 | 217 | 0 |
| WPAH05 | EU304459.1 | Whitmania pigra | 99.552 | 5.74E-114 | 223 | 0 |
| WPAH06 | EU304459.1 | Whitmania pigra | 100 | 2.07E-113 | 218 | 0 |
| WPAH07 | EU304459.1 | Whitmania pigra | 99.543 | 2.64E-112 | 219 | 1 |
| WPAH08 | EU304459.1 | Whitmania pigra | 99.543 | 2.67E-112 | 219 | 1 |
| WPAH09 | EU304459.1 | Whitmania pigra | 99.543 | 2.67E-112 | 219 | 1 |
| WPAH10 | EU304459.1 | Whitmania pigra | 100 | 5.76E-114 | 219 | 0 |
| WPAH11 | EU304459.1 | Whitmania pigra | 100 | 5.76E-114 | 219 | 0 |
| WPAH12 | EU304459.1 | Whitmania pigra | 99.103 | 2.68E-112 | 223 | 0 |
| WPAH13 | EU304459.1 | Whitmania pigra | 100 | 5.74E-114 | 219 | 0 |
| WPAH14 | EU304459.1 | Whitmania pigra | 100 | 5.71E-114 | 219 | 0 |
| WPAH15 | EU304459.1 | Whitmania pigra | 100 | 5.68E-114 | 219 | 0 |
| WPAH16 | EU304459.1 | Whitmania pigra | 100 | 5.71E-114 | 219 | 0 |
| WPAH17 | EU304459.1 | Whitmania pigra | 100 | 5.65E-114 | 219 | 0 |
| WPAH18 | EU304459.1 | Whitmania pigra | 100 | 5.65E-114 | 219 | 0 |
| WPAH19 | EU304459.1 | Whitmania pigra | 100 | 5.68E-114 | 219 | 0 |
| WPAH20 | EU304459.1 | Whitmania pigra | 100 | 5.76E-114 | 219 | 0 |
| WPAH21 | EU304459.1 | Whitmania pigra | 98.655 | 1.25E-110 | 223 | 2 |
| WPAH22 | EU304459.1 | Whitmania pigra | 100 | 5.71E-114 | 219 | 0 |
| WPAH23 | EU304459.1 | Whitmania pigra | 100 | 2.05E-113 | 218 | 0 |
| WPAH24 | EU304459.1 | Whitmania pigra | 100 | 1.58E-114 | 220 | 0 |
| WPAH25 | EU304459.1 | Whitmania pigra | 100 | 5.65E-114 | 219 | 0 |
| WPAH26 | EU304459.1 | Whitmania pigra | 100 | 5.71E-114 | 219 | 0 |
| WPAH27 | EU304459.1 | Whitmania pigra | 97.717 | 1.24E-105 | 219 | 5 |
| WPAH28 | EU304459.1 | Whitmania pigra | 99.545 | 7.35E-113 | 220 | 1 |
| WPAH29 | EU304459.1 | Whitmania pigra | 100 | 5.71E-114 | 219 | 0 |
| WPAH30 | EU304459.1 | Whitmania pigra | 100 | 5.68E-114 | 219 | 0 |
| WPAH31 | EU304459.1 | Whitmania pigra | 99.103 | 7.46E-113 | 223 | 2 |
| WPAH32 | EU304459.1 | Whitmania pigra | 100 | 4.41E-115 | 221 | 0 |
| WPDL01 | EU304459.1 | Whitmania pigra | 99.556 | 4.46E-115 | 225 | 0 |
| WPDL02 | EU304459.1 | Whitmania pigra | 100 | 5.68E-114 | 219 | 0 |
| WPHLJ01 | EU304459.1 | Whitmania pigra | 100 | 5.71E-114 | 219 | 0 |
| WPHLJ02 | EU304459.1 | Whitmania pigra | 96.875 | 5.96E-104 | 224 | 4 |
| WPHN01 | EU304459.1 | Whitmania pigra | 100 | 5.74E-114 | 219 | 0 |
| WPHN02 | EU304459.1 | Whitmania pigra | 100 | 5.68E-114 | 219 | 0 |
| WPJS01 | EU304459.1 | Whitmania pigra | 100 | 5.74E-114 | 219 | 0 |
| WPJS02 | EU304459.1 | Whitmania pigra | 99.543 | 2.64E-112 | 219 | 1 |
| WPJS03 | EU304459.1 | Whitmania pigra | 99.554 | 1.61E-114 | 224 | 0 |
| WPJS04 | EU304459.1 | Whitmania pigra | 100 | 1.58E-114 | 220 | 0 |
| WPJS05 | EU304459.1 | Whitmania pigra | 100 | 5.74E-114 | 219 | 0 |
| WPJS06 | EU304459.1 | Whitmania pigra | 99.548 | 2.05E-113 | 221 | 1 |
| WPJX | EU304459.1 | Whitmania pigra | 99.554 | 1.59E-114 | 224 | 0 |
| WPKS01 | EU304459.1 | Whitmania pigra | 100 | 5.71E-114 | 219 | 0 |
| WPKS02 | EU304459.1 | Whitmania pigra | 99.552 | 5.74E-114 | 223 | 0 |
| WPKS03 | EU304459.1 | Whitmania pigra | 100 | 5.68E-114 | 219 | 0 |
| WPKS04 | EU304459.1 | Whitmania pigra | 100 | 5.68E-114 | 219 | 0 |
| WPKS05 | EU304459.1 | Whitmania pigra | 99.552 | 5.79E-114 | 223 | 0 |
| WPKS06 | EU304459.1 | Whitmania pigra | 100 | 2.01E-113 | 218 | 0 |
| WPSD01 | EU304459.1 | Whitmania pigra | 100 | 5.68E-114 | 219 | 0 |
| WPSD02 | EU304459.1 | Whitmania pigra | 99.543 | 2.72E-112 | 219 | 1 |
| WPSD03 | EU304459.1 | Whitmania pigra | 99.543 | 2.67E-112 | 219 | 1 |
| WPSD04 | MF358688.1 | Erpobdella japonica | 99.07 | 1.99E-108 | 215 | 2 |
| WPSD05 | MF358688.1 | Erpobdella japonica | 99.479 | 2.31E-97 | 192 | 1 |
| WPSD06 | MF358688.1 | Erpobdella japonica | 99.057 | 9.10E-107 | 212 | 2 |
| WPSH | MF358688.1 | Erpobdella japonica | 99.057 | 9.10E-107 | 212 | 2 |
| WPZJ01 | MF358688.1 | Erpobdella japonica | 99.074 | 5.55E-109 | 216 | 2 |
| WPZJ02 | MF358688.1 | Erpobdella japonica | 99.065 | 7.14E-108 | 214 | 2 |
| WPZJ03 | CM079106.1 | Hirudo tianjinensis | 99.541 | 9.74E-112 | 218 | 1 |
| WPZJ04 | CM079106.1 | Hirudo tianjinensis | 100 | 1.63E-114 | 220 | 0 |
| WPZJ05 | CM079106.1 | Hirudo tianjinensis | 99.548 | 7.38E-113 | 221 | 0 |
| WPZJ06 | KC667144.1 | Hirudo nipponia | 99.548 | 7.46E-113 | 221 | 0 |
| WPZJ07 | CM079106.1 | Hirudo tianjinensis | 99.095 | 3.60E-111 | 221 | 1 |
| WPZJ08 | KC667144.1 | Hirudo nipponia | 99.087 | 1.27E-110 | 219 | 2 |
| WPZJ09 | CM079106.1 | Hirudo tianjinensis | 99.552 | 1.60E-114 | 223 | 1 |
| WPZJ10 | CM079106.1 | Hirudo tianjinensis | 99.548 | 7.49E-113 | 221 | 0 |
| YP2 | CM079106.1 | Hirudo tianjinensis | 99.548 | 7.57E-113 | 221 | 0 |
| YP3 | CM079106.1 | Hirudo tianjinensis | 100 | 1.24E-115 | 222 | 0 |
| YP4 | CM079106.1 | Hirudo tianjinensis | 100 | 1.61E-114 | 220 | 0 |
| YP5 | CM079106.1 | Hirudo tianjinensis | 100 | 4.50E-115 | 221 | 0 |
| YP6 | MZ507570.1 | Hirudo nipponia | 100 | 1.23E-115 | 222 | 0 |
| YP7 | MZ507570.1 | Hirudo nipponia | 99.556 | 1.26E-115 | 225 | 1 |

Supplementary Table S3 ASV table of all samples.

| ASV_ID | CPM9 | CPM12 | CPM13 | CPM14 | CPM3 | CPM5 | CPM8 | HUN | Blast result |
| --- | --- | --- | --- | --- | --- | --- | --- | --- | --- |
| ASV_1 | 16814 | 46035 | 139 | 173 | 41654 | 79 | 4427 | 24930 | Whitmania pigra |
| ASV_2 | 16375 | 61 | 29 | 29 | 889 | 35856 | 118 | 23045 | *Erpobdella* japonica |
| ASV_3 | 97 | 53 | 27849 | 24361 | 121 | 40 | 52 | 0 | Hirudo nipponia |
| ASV_4 | 0 | 73 | 15412 | 30407 | 0 | 0 | 74 | 0 | Hirudo nipponia |
| ASV_5 | 21 | 53 | 4285 | 246 | 27 | 12 | 32878 | 1854 | Manillensis manillensis |
| ASV_6 | 0 | 23 | 0 | 0 | 145 | 0 | 0 | 29501 | Whitmania laevis |
| ASV_7 | 5330 | 0 | 0 | 0 | 125 | 13270 | 24 | 0 | *Erpobdella* japonica |
| ASV_8 | 1085 | 8340 | 0 | 0 | 3774 | 0 | 130 | 0 | Whitmania pigra |
| ASV_9 | 1877 | 605 | 0 | 12 | 8359 | 52 | 60 | 0 | Whitmania pigra |
| ASV_10 | 0 | 0 | 3448 | 6244 | 0 | 0 | 0 | 0 | Hirudo nipponia |
| ASV_11 | 3952 | 0 | 0 | 0 | 0 | 3794 | 0 | 0 | *Erpobdella* japonica |
| ASV_12 | 921 | 3025 | 0 | 0 | 1909 | 0 | 394 | 0 | Whitmania pigra |
| ASV_13 | 502 | 2966 | 0 | 0 | 2161 | 0 | 256 | 0 | Whitmania pigra |
| ASV_14 | 0 | 0 | 2004 | 3423 | 0 | 0 | 0 | 0 | Hirudo nipponia |
| ASV_15 | 290 | 146 | 60 | 48 | 1237 | 0 | 3011 | 0 | Whitmania laevis |
| ASV_16 | 11 | 0 | 7 | 9 | 0 | 12 | 4601 | 0 | unknown |
| ASV_17 | 1028 | 0 | 0 | 0 | 0 | 3517 | 0 | 0 | *Erpobdella* japonica |
| ASV_18 | 0 | 0 | 1656 | 2177 | 0 | 0 | 0 | 0 | Hirudo nipponia |
| ASV_19 | 807 | 0 | 0 | 0 | 246 | 2286 | 0 | 0 | *Erpobdella* japonica |
| ASV_20 | 0 | 0 | 0 | 0 | 0 | 0 | 0 | 2472 | Hirudo nipponia |
| ASV_21 | 156 | 1204 | 0 | 0 | 779 | 0 | 0 | 0 | Whitmania pigra |
| ASV_22 | 899 | 0 | 0 | 0 | 5 | 0 | 1036 | 0 | unknown |
| ASV_23 | 403 | 0 | 0 | 0 | 0 | 1369 | 0 | 0 | *Erpobdella* japonica |
| ASV_24 | 0 | 0 | 0 | 0 | 0 | 0 | 1088 | 0 | Barbronia weberi |
| ASV_25 | 0 | 0 | 328 | 559 | 0 | 0 | 0 | 0 | Hirudo nipponia |
| ASV_26 | 282 | 0 | 0 | 0 | 0 | 567 | 0 | 0 | *Erpobdella* japonica |
| ASV_27 | 0 | 0 | 0 | 0 | 0 | 0 | 712 | 0 | Barbronia weberi |
| ASV_28 | 0 | 0 | 0 | 665 | 0 | 0 | 0 | 0 | Hirudo nipponia |
| ASV_29 | 0 | 0 | 0 | 0 | 0 | 0 | 664 | 0 | Whitmania laevis |
| ASV_30 | 0 | 0 | 0 | 0 | 0 | 0 | 611 | 0 | unknown |
| ASV_31 | 0 | 0 | 269 | 335 | 0 | 0 | 0 | 0 | Hirudo nipponia |
| ASV_32 | 190 | 0 | 0 | 0 | 0 | 0 | 390 | 0 | unknown |
| ASV_33 | 0 | 0 | 0 | 571 | 0 | 0 | 0 | 0 | Hirudo nipponia |
| ASV_34 | 74 | 0 | 0 | 0 | 0 | 492 | 0 | 0 | *Erpobdella* japonica |
| ASV_35 | 0 | 0 | 0 | 0 | 0 | 0 | 563 | 0 | unknown |
| ASV_36 | 0 | 0 | 0 | 0 | 0 | 0 | 0 | 549 | Hirudo nipponia |
| ASV_37 | 0 | 0 | 0 | 0 | 0 | 0 | 482 | 0 | Barbronia weberi |
| ASV_38 | 0 | 0 | 0 | 0 | 0 | 0 | 452 | 0 | unknown |
| ASV_39 | 0 | 0 | 446 | 0 | 0 | 0 | 0 | 0 | Hirudo nipponia |
| ASV_40 | 222 | 0 | 0 | 0 | 0 | 222 | 0 | 0 | *Erpobdella* japonica |
| ASV_41 | 83 | 0 | 0 | 0 | 359 | 0 | 0 | 0 | Whitmania laevis |
| ASV_42 | 0 | 0 | 0 | 0 | 0 | 442 | 0 | 0 | *Erpobdella* japonica |
| ASV_43 | 74 | 162 | 0 | 0 | 156 | 0 | 0 | 0 | Whitmania pigra |
| ASV_44 | 52 | 0 | 0 | 0 | 315 | 0 | 0 | 0 | Whitmania laevis |
| ASV_45 | 0 | 0 | 0 | 0 | 0 | 0 | 340 | 0 | unknown |
| ASV_46 | 0 | 0 | 0 | 0 | 0 | 0 | 314 | 0 | Barbronia weberi |
| ASV_47 | 13 | 0 | 0 | 0 | 0 | 0 | 295 | 0 | unknown |
| ASV_48 | 47 | 0 | 0 | 0 | 0 | 252 | 0 | 0 | *Erpobdella* japonica |
| ASV_49 | 0 | 0 | 0 | 0 | 0 | 297 | 0 | 0 | *Erpobdella* japonica |
| ASV_50 | 0 | 0 | 286 | 0 | 0 | 0 | 0 | 0 | Hirudo nipponia |
| ASV_51 | 2 | 3 | 201 | 0 | 0 | 0 | 77 | 0 | Hirudo medicinalis |
| ASV_52 | 264 | 0 | 0 | 0 | 0 | 0 | 5 | 0 | unknown |
| ASV_53 | 0 | 0 | 0 | 0 | 0 | 0 | 245 | 0 | unknown |
| ASV_54 | 0 | 0 | 237 | 0 | 0 | 0 | 0 | 0 | Hirudo medicinalis |
| ASV_55 | 0 | 0 | 158 | 75 | 0 | 0 | 0 | 0 | Hirudo nipponia |
| ASV_56 | 0 | 0 | 0 | 0 | 4 | 0 | 227 | 0 | Barbronia weberi |
| ASV_57 | 0 | 0 | 0 | 0 | 0 | 229 | 0 | 0 | *Erpobdella* japonica |
| ASV_58 | 56 | 0 | 0 | 0 | 0 | 172 | 0 | 0 | *Erpobdella* japonica |
| ASV_59 | 0 | 0 | 0 | 0 | 0 | 0 | 228 | 0 | Barbronia weberi |
| ASV_60 | 0 | 0 | 0 | 0 | 0 | 0 | 225 | 0 | Barbronia weberi |
| ASV_61 | 0 | 0 | 0 | 0 | 221 | 0 | 0 | 0 | Whitmania pigra |
| ASV_62 | 0 | 0 | 0 | 0 | 0 | 0 | 220 | 0 | unknown |
| ASV_63 | 31 | 0 | 0 | 0 | 0 | 185 | 0 | 0 | *Erpobdella* japonica |
| ASV_64 | 82 | 0 | 0 | 0 | 0 | 0 | 133 | 0 | unknown |
| ASV_65 | 0 | 0 | 0 | 0 | 192 | 0 | 0 | 0 | Whitmania pigra |
| ASV_66 | 190 | 0 | 0 | 0 | 0 | 0 | 0 | 0 | *Erpobdella* japonica |
| ASV_67 | 19 | 46 | 0 | 0 | 30 | 0 | 36 | 56 | Whitmania pigra |
| ASV_68 | 0 | 0 | 0 | 0 | 0 | 0 | 186 | 0 | Barbronia weberi |
| ASV_69 | 0 | 0 | 0 | 0 | 0 | 179 | 0 | 0 | *Erpobdella* japonica |
| ASV_70 | 0 | 0 | 0 | 0 | 0 | 0 | 175 | 0 | Barbronia weberi |
| ASV_71 | 15 | 0 | 0 | 0 | 49 | 0 | 99 | 0 | Whitmania acranulata |
| ASV_72 | 0 | 0 | 0 | 0 | 0 | 0 | 161 | 0 | Barbronia weberi |
| ASV_73 | 0 | 0 | 0 | 0 | 0 | 0 | 155 | 0 | unknown |
| ASV_74 | 0 | 0 | 93 | 0 | 0 | 0 | 55 | 0 | Hirudo medicinalis |
| ASV_75 | 0 | 0 | 0 | 0 | 0 | 0 | 139 | 0 | unknown |
| ASV_76 | 0 | 0 | 0 | 0 | 137 | 0 | 0 | 0 | Whitmania pigra |
| ASV_77 | 6 | 0 | 0 | 0 | 0 | 0 | 125 | 0 | unknown |
| ASV_78 | 0 | 0 | 0 | 0 | 0 | 0 | 125 | 0 | unknown |
| ASV_79 | 88 | 0 | 0 | 0 | 8 | 24 | 0 | 0 | Haemopis sanguisuga |
| ASV_80 | 0 | 0 | 0 | 0 | 0 | 0 | 119 | 0 | unknown |
| ASV_81 | 69 | 0 | 2 | 0 | 0 | 0 | 47 | 0 | unknown |
| ASV_82 | 0 | 0 | 0 | 0 | 0 | 0 | 112 | 0 | Barbronia weberi |
| ASV_83 | 106 | 0 | 0 | 0 | 0 | 0 | 0 | 0 | *Erpobdella* japonica |
| ASV_84 | 0 | 0 | 36 | 0 | 0 | 0 | 64 | 0 | Manillensis manillensis |
| ASV_85 | 0 | 0 | 0 | 0 | 0 | 0 | 89 | 0 | Barbronia weberi |
| ASV_86 | 0 | 0 | 0 | 0 | 0 | 0 | 79 | 0 | Whitmania laevis |
| ASV_87 | 0 | 0 | 0 | 0 | 0 | 0 | 75 | 0 | Barbronia weberi |
| ASV_88 | 0 | 0 | 0 | 0 | 0 | 0 | 67 | 0 | Manillensis manillensis |
| ASV_89 | 0 | 0 | 0 | 0 | 0 | 0 | 0 | 66 | *Erpobdella* japonica |
| ASV_90 | 64 | 0 | 0 | 0 | 0 | 0 | 0 | 0 | Whitmania pigra |
| ASV_91 | 0 | 0 | 0 | 0 | 0 | 0 | 62 | 0 | Barbronia weberi |
| ASV_92 | 0 | 0 | 61 | 0 | 0 | 0 | 0 | 0 | Hirudo nipponia |
| ASV_93 | 44 | 0 | 0 | 0 | 0 | 0 | 16 | 0 | unknown |
| ASV_94 | 0 | 0 | 0 | 0 | 59 | 0 | 0 | 0 | Hirudo nipponia |
| ASV_95 | 0 | 0 | 0 | 0 | 0 | 0 | 59 | 0 | Barbronia weberi |
| ASV_96 | 36 | 0 | 0 | 0 | 0 | 0 | 22 | 0 | unknown |
| ASV_97 | 0 | 0 | 0 | 0 | 0 | 0 | 57 | 0 | Whitmania laevis |
| ASV_98 | 0 | 0 | 0 | 0 | 0 | 0 | 57 | 0 | Whitmania laevis |
| ASV_99 | 0 | 0 | 0 | 0 | 55 | 0 | 0 | 0 | Whitmania pigra |
| ASV_100 | 0 | 0 | 0 | 0 | 0 | 0 | 54 | 0 | Whitmania laevis |
| ASV_101 | 0 | 0 | 29 | 0 | 0 | 0 | 20 | 4 | unknown |
| ASV_102 | 0 | 0 | 0 | 0 | 0 | 0 | 53 | 0 | unknown |
| ASV_103 | 0 | 0 | 0 | 0 | 0 | 0 | 53 | 0 | Barbronia weberi |
| ASV_104 | 0 | 0 | 0 | 0 | 0 | 0 | 52 | 0 | unknown |
| ASV_105 | 0 | 0 | 27 | 9 | 0 | 0 | 13 | 0 | Hirudo medicinalis |
| ASV_106 | 0 | 48 | 0 | 0 | 0 | 0 | 0 | 0 | Hirudo nipponia |
| ASV_107 | 0 | 0 | 0 | 0 | 0 | 48 | 0 | 0 | *Erpobdella* japonica |
| ASV_108 | 0 | 0 | 0 | 0 | 0 | 0 | 47 | 0 | unknown |
| ASV_109 | 0 | 0 | 0 | 0 | 0 | 0 | 44 | 0 | Barbronia weberi |
| ASV_110 | 0 | 0 | 0 | 0 | 0 | 0 | 43 | 0 | unknown |
| ASV_111 | 13 | 0 | 0 | 0 | 0 | 28 | 0 | 0 | *Erpobdella* octoculata |
| ASV_112 | 12 | 0 | 0 | 0 | 0 | 0 | 29 | 0 | unknown |
| ASV_113 | 6 | 0 | 0 | 33 | 0 | 0 | 0 | 0 | Hirudo nipponia |
| ASV_114 | 0 | 0 | 0 | 0 | 0 | 0 | 37 | 0 | *Erpobdella* octoculata |
| ASV_115 | 0 | 0 | 0 | 0 | 0 | 36 | 0 | 0 | Barbronia weberi |
| ASV_116 | 0 | 0 | 0 | 0 | 0 | 0 | 36 | 0 | Barbronia weberi |
| ASV_117 | 0 | 0 | 0 | 0 | 0 | 0 | 33 | 0 | Whitmania acranulata |
| ASV_118 | 27 | 0 | 0 | 0 | 0 | 0 | 5 | 0 | unknown |
| ASV_119 | 0 | 0 | 32 | 0 | 0 | 0 | 0 | 0 | Hirudo nipponia |
| ASV_120 | 0 | 0 | 0 | 0 | 0 | 0 | 32 | 0 | Barbronia weberi |
| ASV_121 | 0 | 0 | 0 | 0 | 0 | 0 | 31 | 0 | Hirudo nipponia |
| ASV_122 | 0 | 0 | 0 | 0 | 0 | 30 | 0 | 0 | *Erpobdella* japonica |
| ASV_123 | 28 | 0 | 0 | 0 | 0 | 0 | 0 | 0 | Mooreobdella quaternaria |
| ASV_124 | 0 | 0 | 0 | 0 | 28 | 0 | 0 | 0 | Haemopis sanguisuga |
| ASV_125 | 0 | 0 | 0 | 0 | 0 | 0 | 26 | 0 | Barbronia weberi |
| ASV_126 | 0 | 0 | 0 | 0 | 0 | 25 | 0 | 0 | *Erpobdella* japonica |
| ASV_127 | 0 | 0 | 0 | 0 | 0 | 0 | 24 | 0 | unknown |
| ASV_128 | 0 | 0 | 0 | 0 | 0 | 0 | 24 | 0 | unknown |
| ASV_129 | 0 | 0 | 0 | 0 | 0 | 0 | 22 | 0 | unknown |
| ASV_130 | 0 | 0 | 0 | 0 | 0 | 0 | 22 | 0 | Manillensis manillensis |
| ASV_131 | 0 | 0 | 0 | 0 | 0 | 0 | 22 | 0 | unknown |
| ASV_132 | 19 | 0 | 0 | 0 | 0 | 0 | 0 | 0 | unknown |
| ASV_133 | 0 | 0 | 0 | 0 | 0 | 19 | 0 | 0 | unknown |
| ASV_134 | 0 | 0 | 0 | 0 | 0 | 0 | 19 | 0 | unknown |
| ASV_135 | 0 | 0 | 0 | 0 | 0 | 0 | 17 | 0 | Barbronia weberi |
| ASV_136 | 0 | 0 | 0 | 0 | 0 | 0 | 17 | 0 | Whitmania laevis |
| ASV_137 | 0 | 0 | 14 | 0 | 0 | 0 | 2 | 0 | Poecilobdella javanica |
| ASV_138 | 0 | 0 | 0 | 0 | 16 | 0 | 0 | 0 | *Erpobdella* japonica |
| ASV_139 | 0 | 0 | 0 | 0 | 0 | 0 | 16 | 0 | Poecilobdella manillensis |
| ASV_140 | 15 | 0 | 0 | 0 | 0 | 0 | 0 | 0 | unknown |
| ASV_141 | 0 | 0 | 0 | 0 | 0 | 0 | 15 | 0 | Barbronia weberi |
| ASV_142 | 0 | 3 | 0 | 0 | 0 | 0 | 11 | 0 | unknown |
| ASV_143 | 0 | 0 | 0 | 0 | 13 | 0 | 0 | 0 | unknown |
| ASV_144 | 0 | 0 | 0 | 0 | 0 | 13 | 0 | 0 | unknown |
| ASV_145 | 0 | 0 | 12 | 0 | 0 | 0 | 0 | 0 | Hirudo nipponia |
| ASV_146 | 0 | 0 | 0 | 0 | 0 | 0 | 11 | 0 | unknown |
| ASV_147 | 0 | 0 | 0 | 0 | 0 | 0 | 10 | 0 | unknown |
| ASV_148 | 2 | 0 | 0 | 0 | 0 | 7 | 0 | 0 | unknown |
| ASV_149 | 0 | 9 | 0 | 0 | 0 | 0 | 0 | 0 | Barbronia weberi |
| ASV_150 | 0 | 0 | 0 | 0 | 9 | 0 | 0 | 0 | unknown |
| ASV_151 | 0 | 0 | 0 | 0 | 0 | 9 | 0 | 0 | Whitmania laevis |
| ASV_152 | 0 | 0 | 0 | 0 | 0 | 8 | 0 | 0 | Barbronia yunnanensis |
| ASV_153 | 0 | 0 | 0 | 0 | 0 | 0 | 8 | 0 | Manillensis manillensis |
| ASV_154 | 0 | 0 | 0 | 0 | 0 | 0 | 8 | 0 | Barbronia weberi |
| ASV_155 | 0 | 0 | 0 | 0 | 0 | 0 | 8 | 0 | Hirudo nipponia |
| ASV_156 | 0 | 0 | 0 | 0 | 0 | 0 | 8 | 0 | unknown |
| ASV_157 | 0 | 0 | 0 | 0 | 0 | 0 | 8 | 0 | unknown |
| ASV_158 | 7 | 0 | 0 | 0 | 0 | 0 | 0 | 0 | unknown |
| ASV_159 | 0 | 0 | 0 | 0 | 7 | 0 | 0 | 0 | Whitmania pigra |
| ASV_160 | 0 | 0 | 0 | 0 | 0 | 0 | 7 | 0 | unknown |
| ASV_161 | 0 | 0 | 0 | 6 | 0 | 0 | 0 | 0 | unknown |
| ASV_162 | 0 | 0 | 0 | 0 | 0 | 0 | 6 | 0 | Barbronia weberi |
| ASV_163 | 0 | 0 | 0 | 0 | 0 | 0 | 6 | 0 | unknown |
| ASV_164 | 0 | 0 | 0 | 0 | 5 | 0 | 0 | 0 | Whitmania laevis |
| ASV_165 | 0 | 0 | 0 | 0 | 0 | 5 | 0 | 0 | *Erpobdella* japonica |
| ASV_166 | 0 | 0 | 0 | 0 | 0 | 0 | 5 | 0 | unknown |
| ASV_167 | 0 | 0 | 0 | 0 | 0 | 0 | 5 | 0 | unknown |
| ASV_168 | 0 | 0 | 0 | 0 | 0 | 0 | 5 | 0 | Hirudo medicinalis |
| ASV_169 | 4 | 0 | 0 | 0 | 0 | 0 | 0 | 0 | unknown |
| ASV_170 | 0 | 0 | 0 | 0 | 0 | 0 | 4 | 0 | unknown |
| ASV_171 | 0 | 0 | 0 | 0 | 0 | 0 | 4 | 0 | unknown |
| ASV_172 | 0 | 0 | 0 | 0 | 0 | 0 | 4 | 0 | unknown |
| ASV_173 | 0 | 0 | 3 | 0 | 0 | 0 | 0 | 0 | Poecilobdella javanica |
| ASV_174 | 0 | 0 | 0 | 3 | 0 | 0 | 0 | 0 | unknown |
| ASV_175 | 0 | 0 | 0 | 0 | 0 | 3 | 0 | 0 | unknown |
| ASV_176 | 0 | 0 | 0 | 0 | 0 | 3 | 0 | 0 | unknown |
| ASV_177 | 0 | 0 | 0 | 0 | 0 | 0 | 3 | 0 | unknown |
| ASV_178 | 2 | 0 | 0 | 0 | 0 | 0 | 0 | 0 | unknown |
| ASV_179 | 2 | 0 | 0 | 0 | 0 | 0 | 0 | 0 | unknown |
| ASV_180 | 2 | 0 | 0 | 0 | 0 | 0 | 0 | 0 | unknown |
| ASV_181 | 2 | 0 | 0 | 0 | 0 | 0 | 0 | 0 | unknown |
| ASV_182 | 2 | 0 | 0 | 0 | 0 | 0 | 0 | 0 | unknown |
| ASV_183 | 0 | 2 | 0 | 0 | 0 | 0 | 0 | 0 | unknown |
| ASV_184 | 0 | 0 | 2 | 0 | 0 | 0 | 0 | 0 | Hirudo nipponia |
| ASV_185 | 0 | 0 | 0 | 0 | 0 | 2 | 0 | 0 | Barbronia weberi |
| ASV_186 | 0 | 0 | 0 | 0 | 0 | 2 | 0 | 0 | *Erpobdella* japonica |
| ASV_187 | 0 | 0 | 0 | 0 | 0 | 0 | 2 | 0 | Poecilobdella manillensis |
| ASV_188 | 0 | 0 | 0 | 0 | 0 | 0 | 2 | 0 | Whitmania laevis |
| ASV_189 | 0 | 0 | 0 | 0 | 0 | 0 | 2 | 0 | unknown |
| ASV_190 | 0 | 0 | 0 | 0 | 0 | 0 | 0 | 2 | Whitmania laevis |

Supplementary Table S4 Percentage distribution and identification results of reads in the mock community

| Blast result | *Whitmania pigra* | *Whitmania laevis* | *Hirudo nipponia* | *Poecilobdella manillensis* | *Erpobdella japonica* | Unknow |
| --- | --- | --- | --- | --- | --- | --- |
| Number of reads | 24,986 | 29,503 | 3021 | 1854 | 23,111 | 4 |
| ASV ID | ASV_1, ASV_67 | ASV_6, ASV_190 | ASV_20, ASV_36 | ASV_5 | ASV_2, ASV_89 | ASV_101 |
| Reads abundance (%) | 30.29 | 35.77 | 3.66 | 2.25 | 28.02 | 0.00 |

Supplementary Table S5 The proportion and preparation method of leech components in CPM

| Sample Number | Name | Number of medicinal ingredients | Proportion of leeche | Preparation method of leech |
| --- | --- | --- | --- | --- |
| 1 | MaiLuoShuTongKeLi | 12 | 13% | powder/extract |
| 2 | XueShuanXinMaiNingJiaoNang | 10 | 25% | powder |
| 3 | Tongxinluo Jiaonang | 12 | / | / |
| 4 | Tiandan Tongluo Jiaonang | 10 | / | extract |
| 5 | Qishen jiaonang | 15 | 11.3% | powder/extract |
| 6 | Gongliuqing pian | 11 | / | extract |
| 7 | Qing nao jiang ya pian | 13 | / | extract |
| 8 | Peiyuan Tongnao Jiaonang | 14 | 16% | powder |
| 9 | Naoxintong capsule | 16 | 6.73% | powder |
| 10 | Qiming keli | 8 | / | extract |
| 11 | Dahuang Zhechong wan | 12 | 4.5% | powder |
| 12 | Naoxuekang jiaonang | 1 | 100% | powder |
| 13 | Maixuekang jiaonang | 1 | 100% | powder |
| 14 | Huoxue tongmai jiaonang | 1 | 100% | powder |
| 15 | Danguixiang keli | 23 | / | extract |

Supplementary Table S6 Percentage distribution of reads and number of ASV in CPM

|  | Sequencing reads of ASV | | | | | | | |
| --- | --- | --- | --- | --- | --- | --- | --- | --- |
|  | CPM9 | CPM12 | CPM13 | CPM14 | CPM3 | CPM5 | CPM8 | HUN |
| Total number of ASV reads | 52,820 | 62,857 | 57,125 | 69,385 | 63,094 | 63,586 | 57,448 | 82,479 |
| Identity number of ASV reads | 51,110 | 62,852 | 57,087 | 69,367 | 63,067 | 63,536 | 47,361 | 82,475 |
| Number of ASV | 56 | 19 | 28 | 20 | 32 | 37 | 105 | 10 |
| Identity (%) | 96.76 | 99.99 | 99.93 | 99.97 | 99.96 | 99.92 | 82.44 | 100.00 |

Supplementary Table S7 Primer tag sequences.

| Sample | Barcode |
| --- | --- |
| CPM9 | ACACGA |
| CPM12 | AACTGG |
| CPM13 | TGGCGC |
| CPM14 | GTTGGC |
| CPM3 | TTCACT |
| CPM5 | CACTCA |
| CPM8 | GGCCTG |
| HUN | ACACTC |
